# Ammonia inhibits energy metabolism in astrocytes in a rapid and glutamate dehydrogenase 2-dependent manner

**DOI:** 10.1242/dmm.047134

**Published:** 2020-11-04

**Authors:** Leonie Drews, Marcel Zimmermann, Philipp Westhoff, Dominik Brilhaus, Rebecca E. Poss, Laura Bergmann, Constanze Wiek, Peter Brenneisen, Roland P. Piekorz, Tabea Mettler-Altmann, Andreas P. M. Weber, Andreas S. Reichert

**Affiliations:** 1 Institute for Biochemistry and Molecular Biology I, Medical Faculty, Heinrich Heine University Düsseldorf, Universitätsstr. 1, 40225 Düsseldorf, Germany; 2 Institute of Plant Biochemistry, Cluster of Excellence on Plant Sciences (CEPLAS), Heinrich Heine University Düsseldorf, Universitätsstr. 1, 40225 Düsseldorf, Germany; 3 Plant Metabolism and Metabolomics Laboratory, Cluster of Excellence on Plant Sciences (CEPLAS), Heinrich Heine University Düsseldorf, Universitätsstr. 1, 40225 Düsseldorf, Germany; 4 Institute for Biochemistry and Molecular Biology II, Medical Faculty, Heinrich Heine University Düsseldorf, Universitätsstr. 1, 40225 Düsseldorf, Germany; 5 Department of Otorhinolaryngology and Head/Neck Surgery (ENT), Medical Faculty, Heinrich Heine University Düsseldorf, Moorenstr. 5, 40225 Düsseldorf, Germany

**Keywords:** Hepatic encephalopathy, Hyperammonemia, Mitochondria, TCA cycle, Glutamate dehydrogenase, Brain energy metabolism

## Abstract

Astrocyte dysfunction is a primary factor in hepatic encephalopathy (HE) impairing neuronal activity under hyperammonemia. In particular, the early events causing ammonia-induced toxicity to astrocytes are not well understood. Using established cellular HE models, we show that mitochondria rapidly undergo fragmentation in a reversible manner upon hyperammonemia. Further, in our analyses, within a timescale of minutes, mitochondrial respiration and glycolysis were hampered, which occurred in a pH-independent manner. Using metabolomics, an accumulation of glucose and numerous amino acids, including branched chain amino acids, was observed. Metabolomic tracking of ^15^N-labeled ammonia showed rapid incorporation of ^15^N into glutamate and glutamate-derived amino acids. Downregulating human *GLUD2* [encoding mitochondrial glutamate dehydrogenase 2 (GDH2)], inhibiting GDH2 activity by SIRT4 overexpression, and supplementing cells with glutamate or glutamine alleviated ammonia-induced inhibition of mitochondrial respiration. Metabolomic tracking of ^13^C-glutamine showed that hyperammonemia can inhibit anaplerosis of tricarboxylic acid (TCA) cycle intermediates. Contrary to its classical anaplerotic role, we show that, under hyperammonemia, GDH2 catalyzes the removal of ammonia by reductive amination of α-ketoglutarate, which efficiently and rapidly inhibits the TCA cycle. Overall, we propose a critical GDH2-dependent mechanism in HE models that helps to remove ammonia, but also impairs energy metabolism in mitochondria rapidly.

## Abstract (precis)

**Summary:** Here, we identified a mechanism relevant for hepatic encephalopathy by which glutamate dehydrogenase 2 impairs energy metabolism in mitochondria very rapidly via inhibiting the TCA cycle in astrocytes.

## INTRODUCTION

Hepatic encephalopathy (HE) is a severe neuropsychiatric disorder caused by hyperammonemia due to acute or chronic liver dysfunction, most commonly liver cirrhosis. Another major cause of HE is portosystemic shunting, leading to the distribution of portal blood without removal of toxins in the liver (Cash et al., 2010). It is estimated that 50-70% of all patients suffering from liver cirrhosis develop minimal HE or HE (Patidar and Bajaj, 2015). Given that more than 600,000 people are estimated to suffer from liver cirrhosis in the USA (Scaglione et al., 2015), this corresponds to ∼300,000 to 420,000 people with HE in the USA alone. The symptoms can vary from mild to severe, ranging from confusion to hepatic coma and death (Ferenci et al., 2002). So far, the only curative treatment is liver transplantation (Larsen et al., 1995). Treatment options to reduce symptoms are manifold, but insufficient, and have often not been subject to randomized control studies (Ferenci, 2017). A major precipitation factor in HE is hyperammonemia resulting from the impaired ability of the liver to eliminate ammonia via the urea cycle (Ferenci, 2017). Ammonia passes the blood-brain barrier (Lockwood et al., 1979, 1980), causing swelling and production of reactive oxygen species (ROS)/reactive nitrogen species in astrocytes (Görg et al., 2013; Norenberg et al., 2005). Increased ROS levels lead to mitochondrial dysfunction, energy failure, formation of the mitochondrial transition pore (Bai et al., 2001; Stewart et al., 2000; Frank et al., 2012) or RNA oxidation (Görg et al., 2008). Several studies demonstrated that hyperammonemia primarily disturbs astrocyte function, resulting in subsequent neurological dysfunction (Görg et al., 2018; Norenberg, 1987). Astrocytes have an essential role in ensuring neuronal function, such as by providing nutrients, recycling the neurotransmitter glutamate (Sontheimer, 1995; Zalc, 1994) and detoxifying ammonia in the brain, which is thought to occur primarily via ammonia fixation by glutamine synthetase (Martinez-Hernandez et al., 1977). However, the molecular mechanism of ammonia-induced neurological impairment and the role of known ammonia detoxification pathways are unclear.

An increase in mitochondrial fission was reported to occur in mice with severe liver damage in the substantia nigra, but not in the prefrontal cortex (Bai et al., 2018). In various HE rat models, it was shown that the activity of respiratory chain complexes was decreased in different brain regions (Boer et al., 2009; Dhanda et al., 2017). Additionally, an impact on the TCA cycle under hyperammonemia has been discussed, given that some TCA cycle enzymes, e.g. pyruvate dehydrogenase and isocitrate dehydrogenase, were reported to be inhibited by ammonia (Katunuma et al., 1966; Zwingmann et al., 2003). The role of the TCA cycle in the pathogenesis of HE is controversially discussed (reviewed in Rama Rao and Norenberg, 2012). Additionally, a recent analysis of the metabolome of cerebrospinal fluid (CSF) of HE patients points towards alterations in metabolic pathways linked to energy metabolism (Weiss et al., 2016). Overall, disturbances in mitochondrial morphology and imbalances in various energy metabolism pathways have been suggested as a consequence of hyperammonemia; however, their possible contributions to the pathogenesis of HE are still unclear. In most HE models, prolonged treatments with ammonia (such as 24 h and beyond) have been used and are quite well studied (Görg et al., 2015; Hazell and Norenberg, 1998; Oenarto et al., 2016). However, the early events upon exposure to high ammonia concentrations are unclear. Here, we focused on effects after short durations of ammonia treatment and immediate outcomes thereof.

The mitochondrial network constantly undergoes fission and fusion events, which are important to maintain mitochondrial quality control (Nunnari et al., 1997; Schäfer and Reichert, 2009). It was shown that different stress conditions, such as heat shock or increased ROS, can trigger fission of mitochondria, leading to a fragmented mitochondrial morphology (Frank et al., 2012; Duvezin-Caubet et al., 2006). Polletta et al. (2015) have shown the appearance of small, round mitochondria in MDA-MB-231 human breast cancer and C2C12 mouse myoblast cells after treatment with NH_4_Cl, among other stressors. Overall, it is not well understood whether and how altered mitochondrial function and energy metabolism could contribute to the pathogenesis of HE. Here, we decided to address the early effect of hyperammonemia on mitochondrial function and energy metabolism using cellular models of HE. We show that ammonia impairs mitochondrial oxidative phosphorylation (OXPHOS) very rapidly and provide several lines of evidence that ammonia is primarily fixed by reductive amination of α-ketoglutarate to generate glutamate, catalyzed by the mitochondrial glutamate dehydrogenase 2 (GDH2). This provides a novel view on the early steps of ammonia-induced toxicity and will likely help to better understand the pathogenesis of HE in future studies.

## RESULTS

### Ammonia induces mitochondrial fragmentation in a rapid and reversible manner

**Fig. 1.**
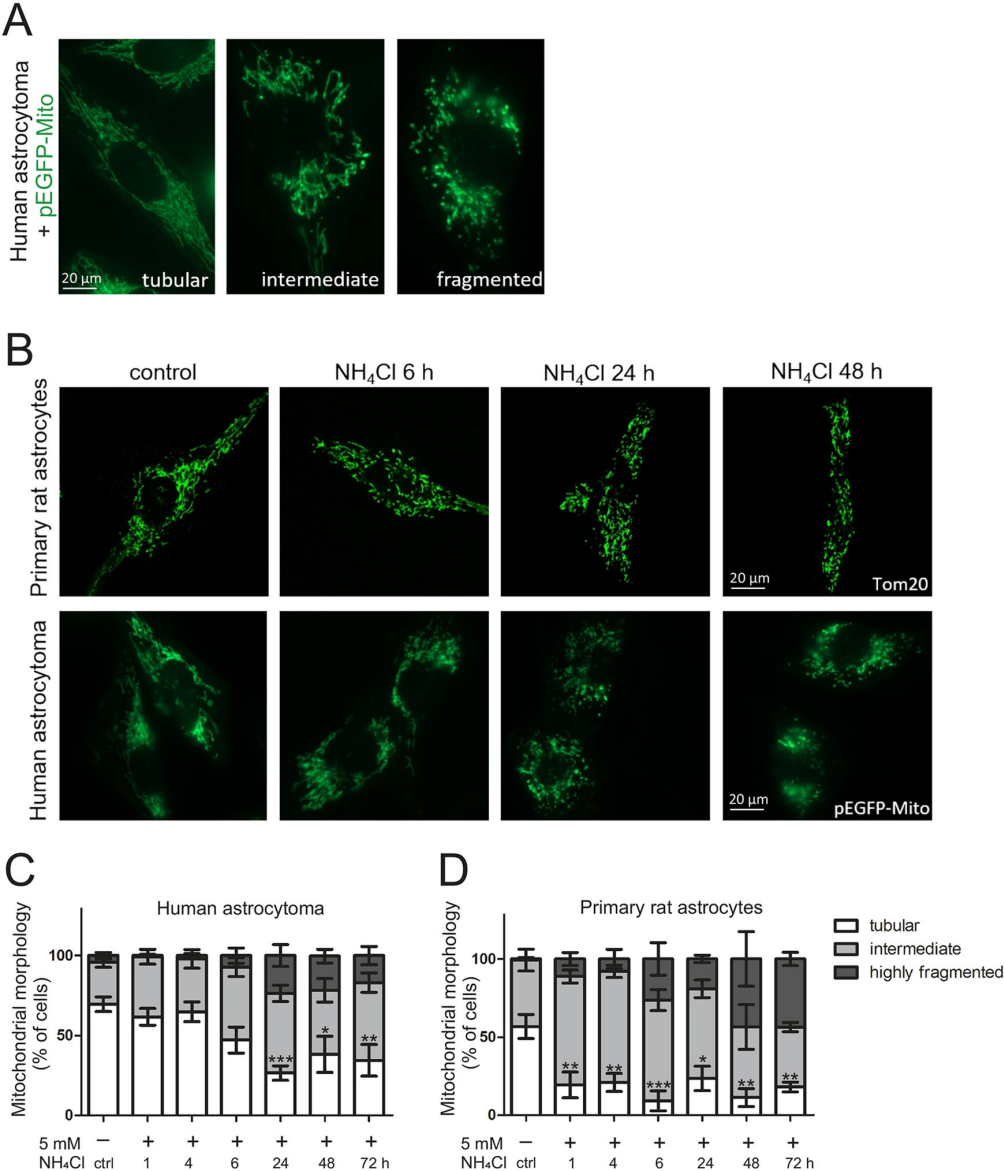
**Mitochondrial morphology is altered by ammonia.** Human astrocytoma cells were transfected with pEGFP-Mito to visualize mitochondria. In primary rat astrocytes, visualization was achieved by immunostaining against Tom20. Cells were treated with 5 mM NH_4_Cl for respective durations. At least 20 pictures were taken per sample, showing approximately three to five cells each. Mitochondria were categorized as tubular, intermediate and fragmented morphological phenotype. (A) Representative images of morphological phenotype characterization in human astrocytoma cells. (B) Changes in morphology of mitochondria after treatment with 5 mM NH_4_Cl in primary rat astrocytes (top row) and human astrocytoma (bottom row). (C,D) Time course of changes in mitochondrial morphology in human astrocytoma (*n*=3-6) (C) and primary rat astrocytes (*n*=3) (D). Data are presented as mean±s.e.m. Statistics: one-way ANOVA with Dunnett's post test (all treatments versus control) for tubular morphology. *P<0.05, **P<0.01, ***P<0.001.

In order to test whether modulation of mitochondrial morphology by ammonia represents an early event in ammonia-induced effects in astrocytes, we transfected the human astrocytoma cell line MOG-G-CCM with pEGFP-Mito, a GFP variant targeting the mitochondrial matrix (Weber et al., 2013). Cells were treated with 5 mM NH_4_Cl for 1-72 h and changes in mitochondrial morphology were quantified using confocal fluorescence microscopy. Similar conditions are used in numerous established *in vitro* HE models (Görg et al., 2015; Warskulat et al., 2002), and a similar ammonia concentration (5.4 mM) was present in the brain of an *in vivo* rat model (Swain et al., 1992). The appearance of cells showing enhanced mild (intermediate) or severe (highly fragmented) mitochondrial fragmentation (Fig. 1A) was evident after 6 h of treatment, compared to controls or to earlier time points (Fig. 1B,C). Mitochondrial fragmentation became more prominent after 24 h, reaching a high steady-state level. Treatment with NH_4_Cl for 48 h and 72 h did not further increase mitochondrial fragmentation (Fig. 1C). To corroborate this in primary cells, we used an established *in vitro* HE model, namely primary rat astrocytes treated with 5 mM NH_4_Cl. In these astrocytes, mitochondrial morphology [visualized by immunostaining against Tom20 (also known as Tomm20)] was rapidly altered by NH_4_Cl (Fig. 1B). The change towards mitochondrial fragmentation was even more rapid as it already became evident after 1 h of ammonia treatment (Fig. 1D). Hence, mitochondrial morphology is altered very rapidly by hyperammonemia and primary rat astrocytes appear to react even faster than human astrocytoma cells. Next, we asked whether mitochondrial fragmentation is reversible upon removal of ammonia. Indeed, we observed in primary rat astrocytes that mitochondrial morphology, which was highly fragmented after 72 h treatment with NH_4_Cl, recovered within 24 h to a highly tubular morphology when the medium was exchanged to fresh medium lacking NH_4_Cl (Fig. S2). We conclude that mitochondrial fragmentation is rapidly induced by hyperammonemia within 1-6 h and is nearly fully reversible within 24 h after the removal of ammonia.

### Ammonia causes an immediate inhibition of mitochondrial respiration in a pH-independent manner

**Fig. 2.**
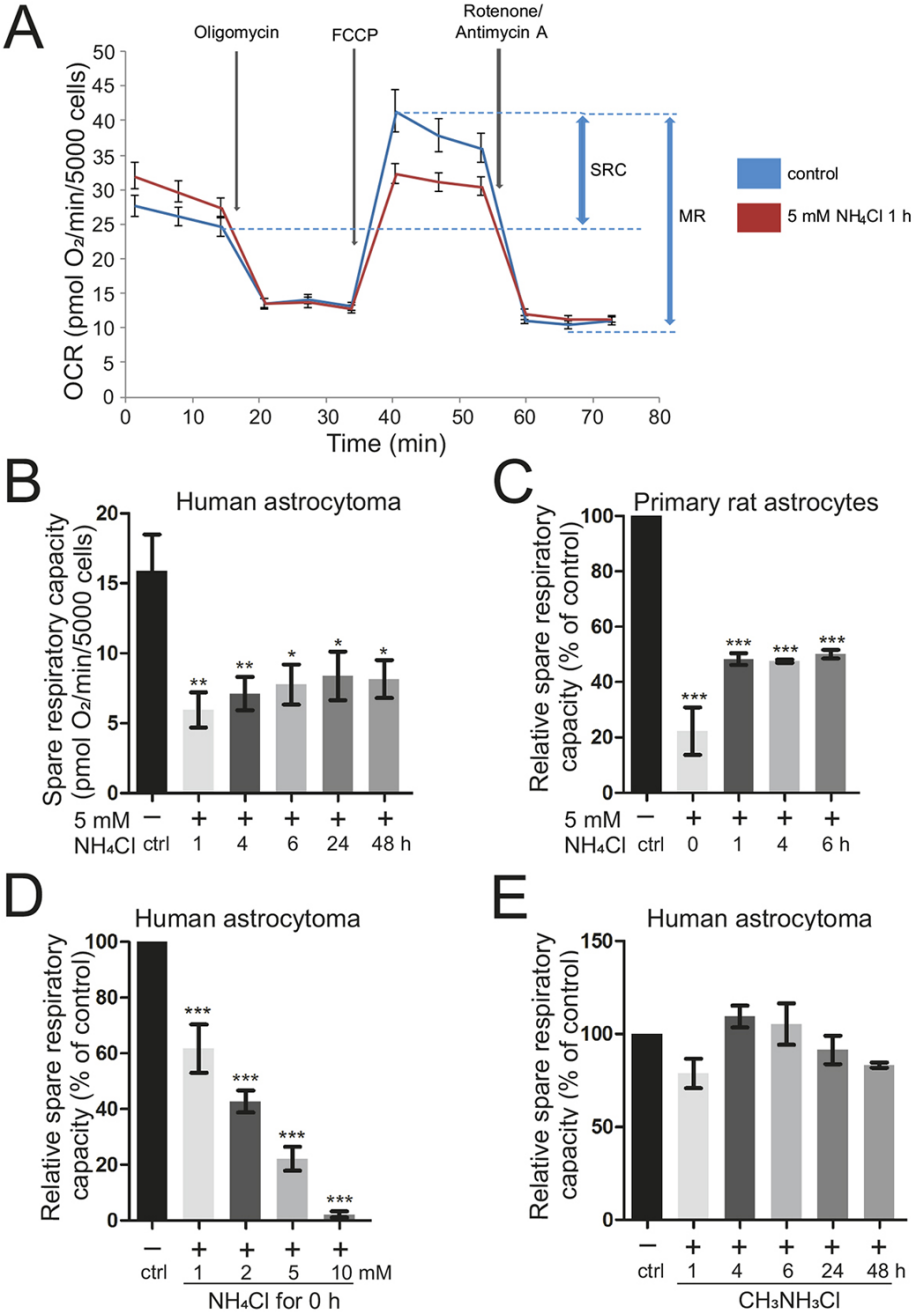
**Mitochondrial respiration is immediately impaired by ammonia in a pH-independent manner.** Oxygen consumption rate (OCR) of human astrocytoma cells and primary rat astrocytes was analyzed in a Seahorse XFe96 Extracellular Flux Analyzer with a Mito Stress Test Kit after treatment with ammonia at the indicated molarity for the indicated duration. (A) Scheme of Seahorse Mito Stress Test with injections. MR, maximal respiration; SRC, spare respiratory capacity. (B) SRC of human astrocytoma cells after 5 mM NH_4_Cl treatment for 1-48 h (*n*=5-7). (C) Relative SRC of primary rat astrocytes after 5 mM NH_4_Cl treatment for 1-6 h and live (0 h) (*n*=3-4). (D) Relative SRC of human astrocytoma cells after live treatment with 1, 2, 5 and 10 mM NH_4_Cl (*n*=3). (E) Relative SRC of human astrocytoma cells after 5 mM CH_3_NH_3_Cl (pH-mimetic) treatment for 1-48 h (*n*=3). Individual biological replicates normalized to control (100%) (C-E). Data are presented as mean±s.e.m. Statistics: one-way ANOVA with Dunnett's post test (all treatments versus control). *P<0.05, **P<0.01, ***P<0.001.

Mitochondrial fragmentation is one of the early hallmarks indicating mitochondrial dysfunction (Frank et al., 2012; Duvezin-Caubet et al., 2006; Jheng et al., 2012). To investigate whether the change in mitochondrial morphology comes hand in hand with mitochondrial dysfunction, we determined the cellular oxygen consumption rate (OCR) in human astrocytoma cells and in primary rat astrocytes after treatment with 5 mM NH_4_Cl for variable time periods. We applied a Mito Stress Test Kit using a Seahorse XFe96 Extracellular Flux Analyzer to determine different parameters of mitochondrial respiration, including spare respiratory capacity and maximal respiration (see Fig. 2A and the ‘Cellular metabolism analysis’ section of the Materials and Methods). These two parameters represent the mitochondrial respiration in a challenged state by uncoupling using carbonyl-cyanide-4-(trifluoromethoxy)-phenylhydrazone (FCCP). In human astrocytoma cells, the spare respiratory capacity (Fig. 2B) and the maximal respiration (Fig. S1A) were significantly reduced after 1 h of ammonia treatment compared to non-treated controls. There was a trend showing a similar effect for basal respiration but this was not statistically significant (Fig. S1E). Prolonged ammonia pretreatments up to 48 h likewise resulted in significant impairment of mitochondrial respiration. It appeared that respiration is more affected with short times of ammonia pretreatment compared to longer times (Fig. 2B; Fig. S1A). To elaborate this further and to check how fast ammonia can affect mitochondrial respiration, we measured the OCR immediately after NH_4_Cl was added. Indeed, this was sufficient to impair mitochondrial respiration as spare respiratory capacity (Fig. 2C) and maximal respiration (Fig. S1B) in primary rat astrocytes were significantly reduced. This reduction without pretreatment (0 h) was even stronger when compared to 1 h, 4 h or 6 h ammonia pretreatment, demonstrating that ammonia has an immediate strong effect on mitochondrial respiration, which moderately decreases with incubation time. We next tested whether this effect is dose dependent. This is indeed the case as concentrations as low as 1 mM NH_4_Cl were sufficient to induce a substantial drop in respiration, which increased with higher concentrations (Fig. 2D; Fig. S1C). Ammonia is a potent base, causing alkalization of extra- and intracellular compartments even under buffering conditions. To analyze whether the observed effect is simply a pH-mediated effect, we repeated the assay using 5 mM CH_3_NH_3_Cl, which cannot be metabolized but acts as a pH mimetic to NH_4_Cl. This did not impair mitochondrial respiration to any significant extent, independent of the duration of pretreatment, except for maximal respiration after 48 h (Fig. 2E; Fig. S1D). This excludes that the observed effect of ammonia results from changes in the pH. We also did not observe any gross changes in the steady-state levels of marker proteins required for OXPHOS that could explain the observed effects (Fig. S9C,D). Moreover, we tested whether removal of ammonia for 1 h is sufficient to restore mitochondrial respiration in human astrocytoma cells that have been pretreated for 1-48 h. One hour in medium without ammonia led to a full recovery of mitochondrial respiration independent of the duration of pretreatment (Fig. S3). This is consistent with our data showing that mitochondrial fragmentation is restored after removal of ammonia from the medium.

### Ammonia impairs glycolysis in astrocytes in a rapid manner

**Fig. 3.**
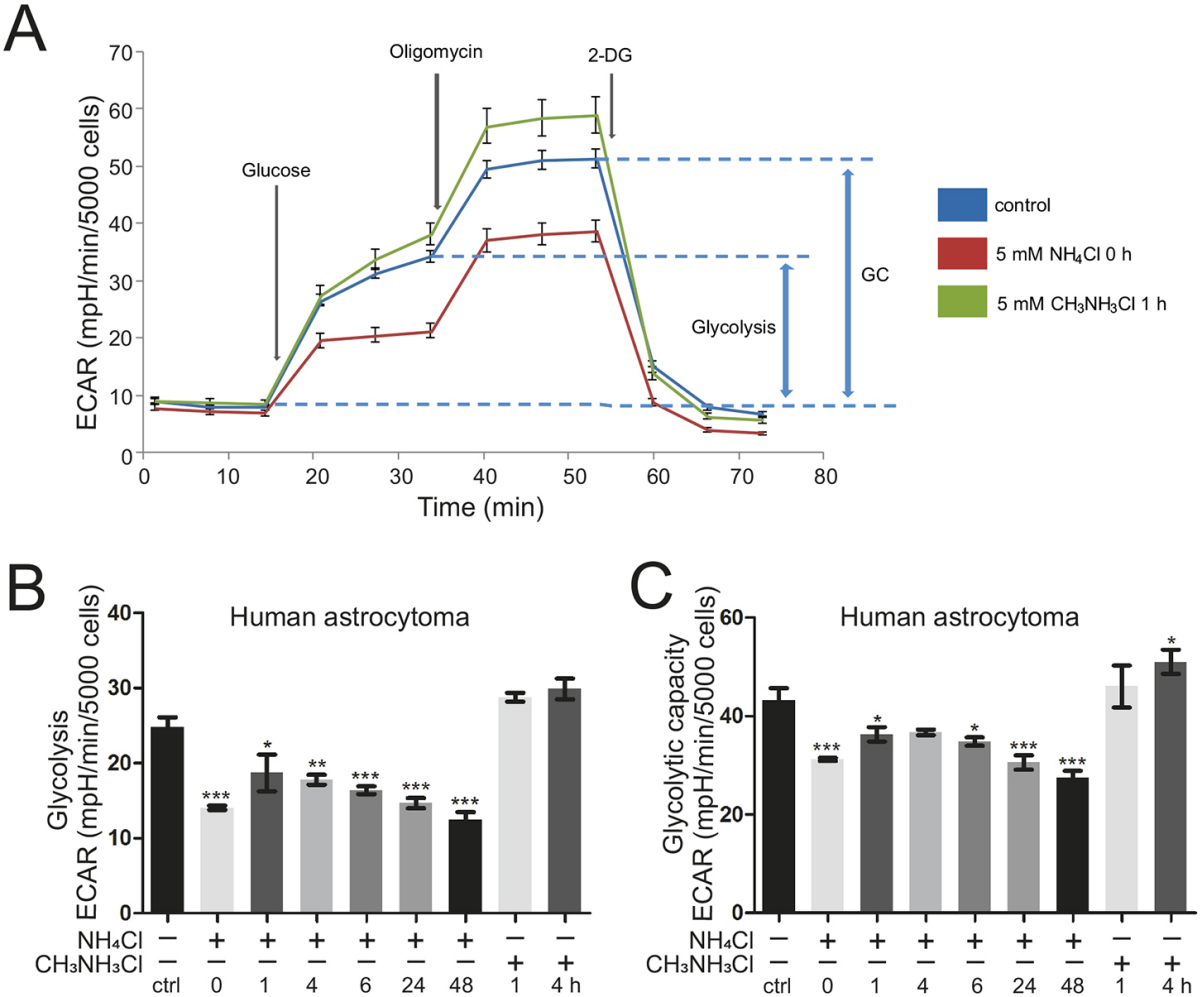
**Ammonia impairs glycolytic function.** Extracellular acidification rate (ECAR) was measured in human astrocytoma cells as a proxy for glycolysis using a Glycolysis Stress Test Kit and Seahorse XFe96 Extracellular Flux Analyzer. (A) Scheme of the Seahorse Glycolysis Stress Test with respective injections indicated. GC, glycolytic capacity; 2-DG, 2-deoxyglucose. (B,C) Glycolysis (B) and glycolytic capacity (C) of human astrocytoma cells after treatment with 5 mM NH_4_Cl for 1-48 h and live (0 h) (*n*=3); treatment with 5 mM pH-mimetic CH_3_NH_3_Cl for 1 h and 4 h (*n*=2). Data are presented as mean±s.e.m. Statistics: one-way ANOVA with Dunnett's post test (all treatments versus control). *P<0.05, **P<0.01, ***P<0.001.

We examined the influence of ammonia on glycolytic flux and glycolytic capacity using a Glycolysis Stress Test with the Seahorse XFe96 Analyzer. Here, the extracellular acidification rate (ECAR) is measured under different conditions and used to estimate glycolysis (see Fig. 3A and the ‘Cellular metabolism analysis’ section of the Materials and Methods). Treatment of human astrocytoma cells with ammonia led to a significant and rapid reduction in both glycolytic flux (Fig. 3B) and glycolytic capacity (Fig. 3C) compared to controls. This was largely independent of the duration of ammonia pretreatment. Treatment with the pH mimetic CH_3_NH_3_Cl did not have any detrimental effect on glycolysis, further corroborating the specific role of ammonia, independent of its property to alter pH. Overall, we conclude that ammonia results in a very rapid, pH-independent impairment of two major bioenergetic metabolic pathways, namely OXPHOS and glycolysis. The observation that both pathways are inhibited by ammonia could be explained by an impairment at the level of the TCA cycle. The latter would not only impair formation of NADH/FADH_2_ used during OXPHOS but also lead to an accumulation of acetyl-CoA and pyruvate. Without a rapid metabolic shift to convert pyruvate to lactate by lactate dehydrogenase, this would inhibit the glycolytic flux via product inhibition. In addition, NAD^+^ is neither regenerated by the latter reaction nor the TCA cycle in sufficient amounts, also explaining reduced glycolysis. Nonetheless, the possibility exists that glycolysis is directly inhibited by ammonia, which we cannot exclude at this stage.

### Targeted metabolomics analysis suggests alteration of the TCA cycle

**Fig. 4.**
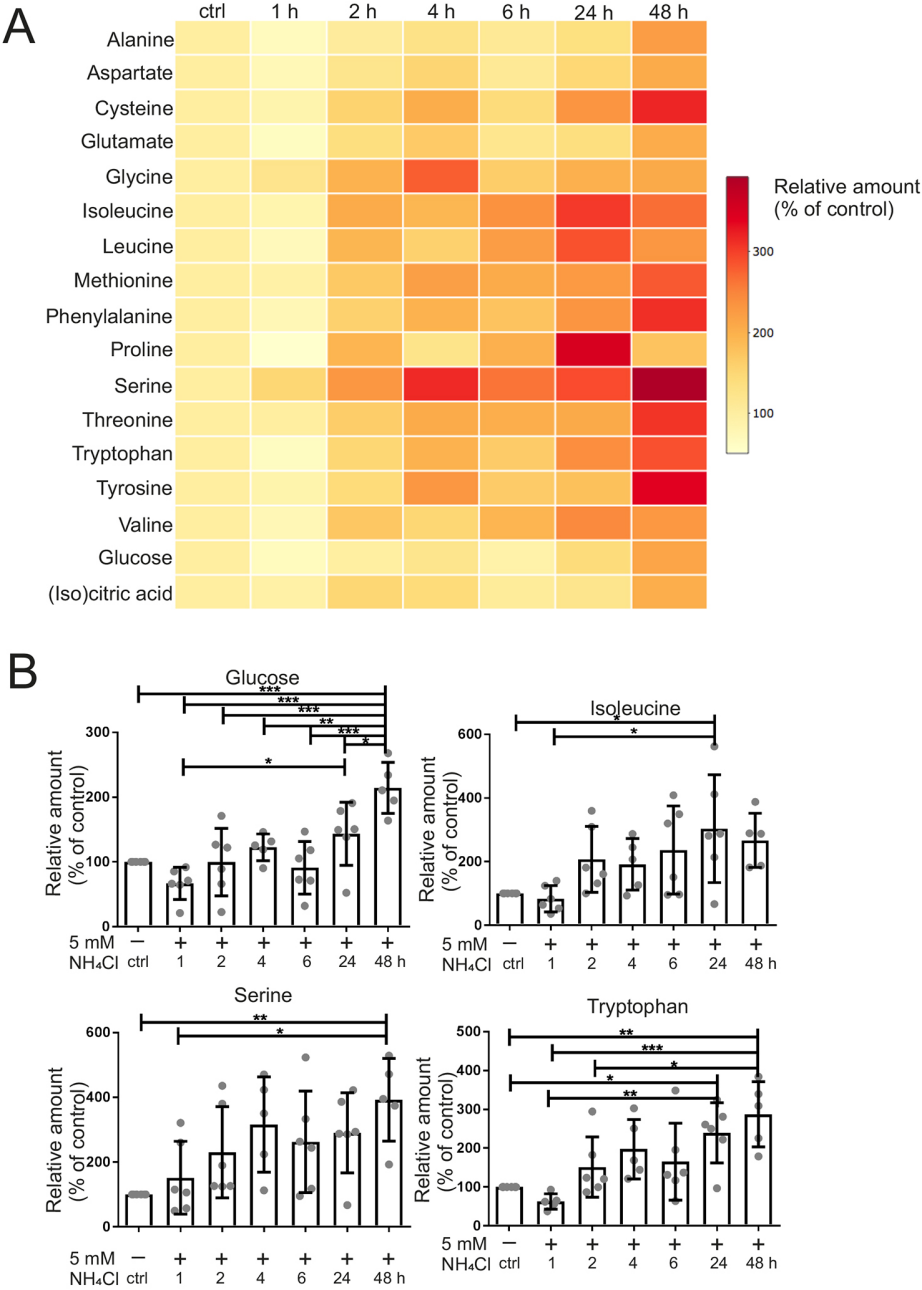
**Ammonia alters energy and amino acid metabolism.** Mass spectrometry for steady-state metabolites was performed in human astrocytoma cells on a GC-QTOF. Treatment was with 5 mM NH_4_Cl for 1-48 h. (A) Heat map showing the relative abundance of amino acids, glucose and (iso)citric acid compared to control (100%) over time. (B) Selected metabolites as indicated from A with additional details. Data are presented as mean±s.d. (*n*=4-6). Statistics: one-way ANOVA with Tukey's post test (all samples versus all samples). *P<0.05, **P<0.01, ***P<0.001.

**Fig. 5.**
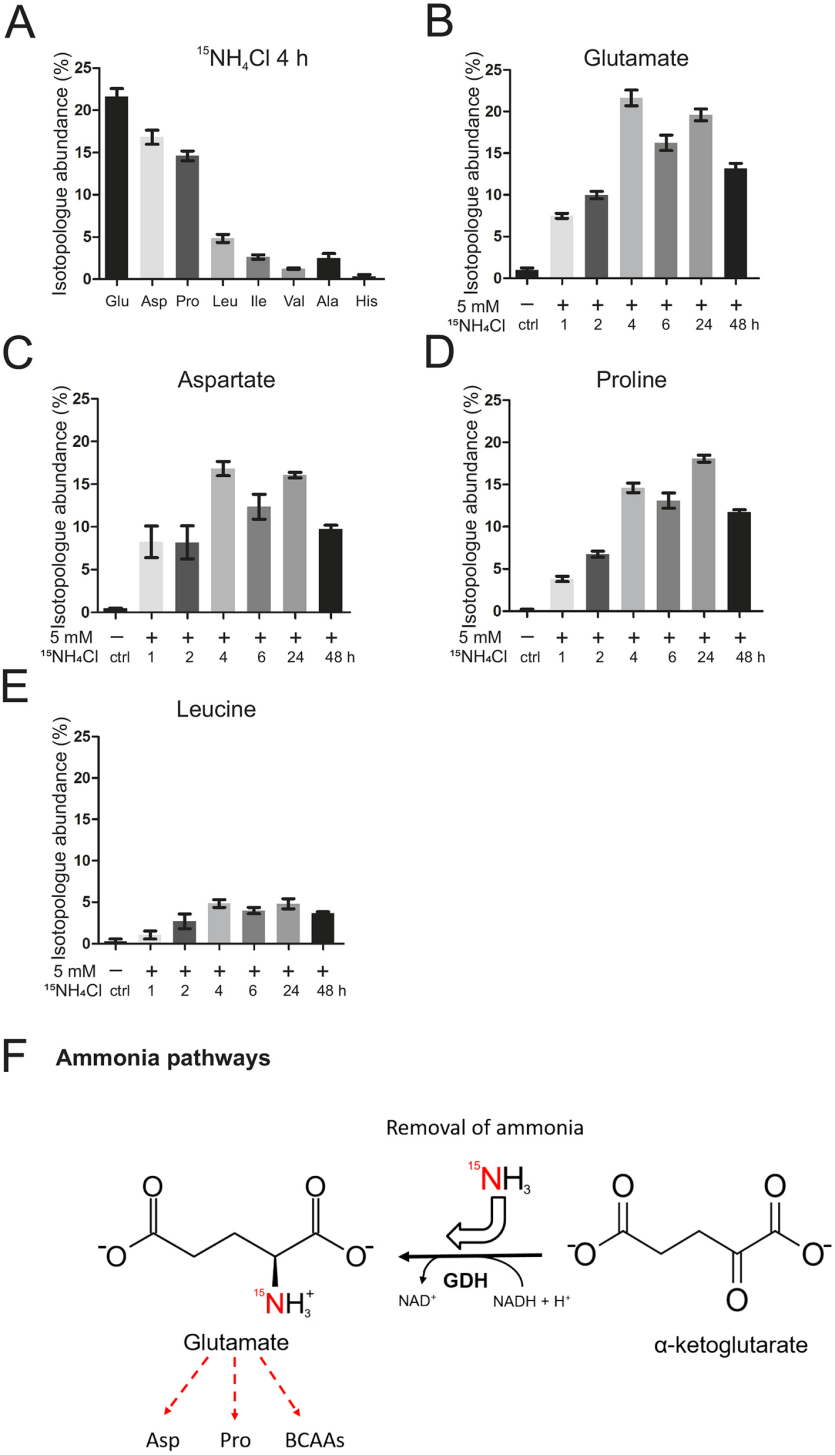
**Ammonia is incorporated into GDH-dependent metabolites.** Mass spectrometry for ammonia flux was performed in human astrocytoma cells on an LC-QTOF. Treatment was with ^15^NH_4_Cl for 1-48 h. (A) Amino acids found to be enriched in ^15^N-isotopologue abundance, representative after 4 h treatment. Values are corrected for natural abundance of heavy isotopes. (B-E) Isotopologue abundance of ^15^N in glutamate (B), aspartate (C), proline (D) and leucine (E) over time. (F) Pathway of ^15^N-ammonia recycling and utilization via glutamate dehydrogenase (GDH) and secondary reactions. Red arrows indicate the path of nitrogen. Data are presented as mean±s.d. (*n*=3). BCAA, branched-chain amino acid.

Ammonia leads to a rapid and drastic change in energy metabolism. To check whether metabolites linked to energy metabolism, including amino acids and TCA cycle intermediates, are altered by ammonia, we treated human astrocytoma cells with 5 mM NH_4_Cl for 1-48 h and subjected them to a targeted metabolomics analysis (Fig. 4A,B; Fig. S4, Tables S1 and S2). We observed a significant increase in the level of isocitric and/or citric acid, which are indistinguishable by the gas chromatography–mass spectrometry (GC-MS) system used here, after 48 h of ammonia treatment (Fig. S4). Moreover, the branched-chain amino acids (BCAAs) isoleucine/leucine/valine were significantly increased after 24 h. Other essential amino acids – methionine, phenylalanine, threonine and tryptophan – were increased after 24 h and/or 48 h, respectively. The non-essential amino acids – alanine, aspartate, cysteine, proline, serine and tyrosine – were increased after 24 h and/or 48 h, whereas glycine showed a significant increase only after 4 h of treatment. We noted a slight improvement in mitochondrial respiration coinciding with the increase in numerous amino acids at late time points (Fig. 2B; Fig. S1A), which could point to a delayed role of these amino acids in a compensatory response. To test the fate of ammonia further, we traced the incorporation of NH4^+^ in human astrocytoma cells using isotopically labeled ^15^NH_4_Cl under the same conditions as before. Using liquid chromatography–mass spectrometry (LC-MS) analysis we found the strongest enrichment of ^15^N-isotopologues for glutamate, followed by aspartate, proline and BCAAs (Fig. 5A-E; Fig. S5, Tables S3 and S4). Of note, a strong enrichment occurred after 1 h, emphasizing a rapid effect of hyperammonemia. Interestingly, the synthesis of glutamate and its downstream metabolites aspartate and proline, as well as the BCAAs, are catalyzed by the enzyme glutamate dehydrogenase (GDH), or downstream reactions, e.g. transaminase reactions, to form BCAAs (Fig. 5F). Overall, these results indicate that mitochondrial glutamate dehydrogenase 2 (GDH2, encoded by *GLUD2*), which catalyzes the reductive amination of α-ketoglutarate and ammonia to glutamate, could be involved in the initial fixation of ammonia in mitochondria.

### Ammonia-induced impairment of mitochondrial respiration depends on GDH2

**Fig. 6.**
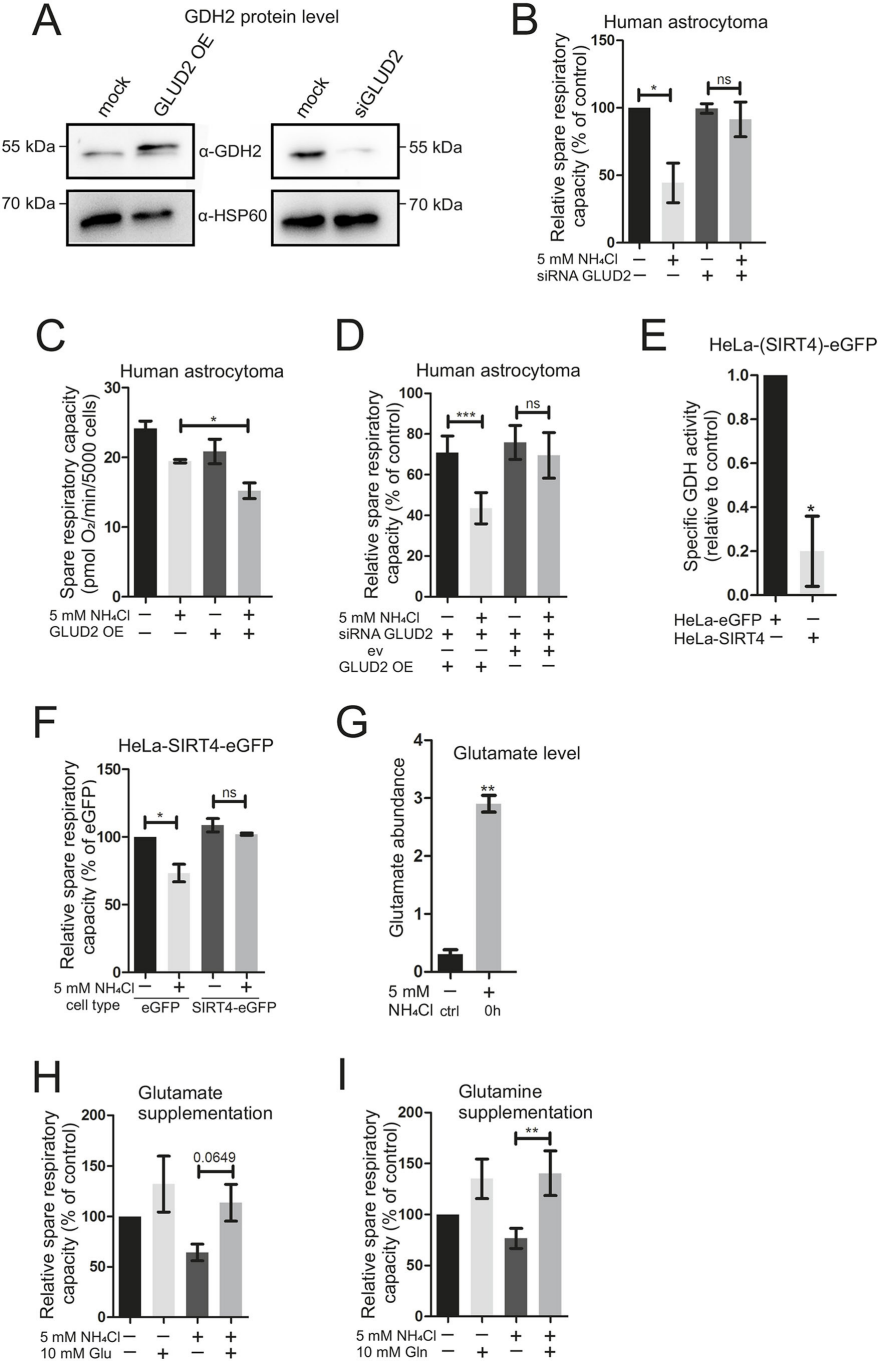
**Knockdown of *GLUD2* and anaplerotic supplementation reverses,** **whereas overexpression exacerbates, the detrimental effect of NH_4_Cl on mitochondrial respiration.** (A) Representative western blot analysis of human astrocytoma cell lysates from *GLUD2* overexpression (48 h) and knockdown (48 h) versus mock samples (transfection reagent only). α-HSP60 was used as a loading control. (B) Oxygen consumption rate (OCR), represented as relative spare respiratory capacity, in human astrocytoma cells was analyzed on a Seahorse XFe96 Extracellular Flux Analyzer with a Mito Stress Test Kit. *GLUD2* knockdown via siRNA transfection for 48 h±5 mM NH_4_Cl treatment for 1 h. Individual biological replicates normalized to control (100%). Statistics: one-sample *t*-test. (C) Spare respiratory capacity of human astrocytoma cells. Treatment with 5 mM NH_4_Cl for 1 h. *GLUD2* overexpression (OE) for 48 h (*n*=5). (D) Relative spare respiratory capacity of human astrocytoma cells. Treatment with 5 mM NH_4_Cl for 1 h. Knockdown of *GLUD2* for 48 h when indicated (siRNA), overexpression of *GLUD2* plasmid (OE) or empty vector (ev) for 48 h when indicated. Individual biological replicates normalized to control (100%, not shown) (*n*=5). (E) GDH activity was measured in HeLa-eGFP cells and HeLa-SIRT4-eGFP cells. GDH activity was determined by a commercial assay detecting NADH production. Values were individually normalized to control and corrected for protein content using Bradford assays. Data are presented as mean±s.e.m. (*n*=3). Statistics: one-sample *t*-test. (F) Relative spare respiratory capacity (%) measured with a Seahorse Analyzer using a Mito Stress Test Kit. HeLa-eGFP and HeLa-SIRT4-eGFP cells treated with 5 mM NH_4_Cl for 1 h and respective controls. Individual biological replicates normalized to HeLa-eGFP cells (100%) (*n*=3). (G) Targeted metabolomics quantification of glutamate in human astrocytoma cells immediately after treatment with 5 mM NH_4_Cl (0 h). Measurement was performed on a GC-QTOF (*n*=3). (H,I) Human astrocytoma cells were subject to Mito Stress Kit measurement on Seahorse XFe96 Extracellular Flux Analyzer. Treatment with (+) or without (−) 10 mM respective compound [H, glutamate (*n*=4-5); I, glutamine (*n*=5)] with (+) or without (−) 5 mM NH_4_Cl for 1 h. Relative spare respiratory capacity is represented by OCR. Individual biological replicates normalized to control (100%). Data are presented as mean±s.e.m. Statistics: one-tailed Student's *t*-test comparing two groups. *P<0.05, **P<0.01, ***P<0.001; ns, not significant.

To test this hypothesis, GDH2 was downregulated in human astrocytoma cells by targeting *GLUD2* gene expression using small interfering RNA (siRNA). Knockdowns were validated via western blotting (Fig. 6A; Fig. S6A), quantitative PCR (Fig. S6B) and determination of specific GDH activity (Fig. S10B). Cells depleted for GDH2 and corresponding controls were subjected to analysis of OCR using the Mito Stress Test. Depletion of GDH2 prevented the reduction of mitochondrial respiration upon treatment for 1 h with ammonia (Fig. 6B), suggesting that GDH2 is specifically required for the observed rapid ammonia-induced impairment of mitochondrial respiration. To investigate this further, GDH2 was overexpressed and subjected to OCR measurements. Overexpression of GDH2 was confirmed via western blotting (Fig. 6A; Fig. S6C) and determination of specific GDH activity (Fig. S10C). An additional band of higher molecular weight (Fig. 6A) can be attributed to a fraction of non-imported GDH2 precursor protein due to the overexpression. Treatment of GDH2-overexpressing cells with 5 mM NH_4_Cl for 1 h exacerbated the decrease in mitochondrial respiration observed before (Fig. 6C). To corroborate this, and to exclude off-target effects of the *GLUD2* siRNA used earlier, we also overexpressed GDH2 in the presence of the *GLUD2* siRNA. It should be noted that the sequence of *GLUD2* encoded on the plasmid is resistant to the used siRNA. Indeed, overexpression of GDH2 from a plasmid restored the sensitivity of human astrocytoma cells depleted for endogenous GDH2 to ammonia, excluding off-target effects of the *GLUD2* siRNA, and further supported that GDH2 is specifically required for ammonia to impair mitochondrial respiration (Fig. 6D). Moreover, in accordance with the GDH2 knockdown results presented earlier (Fig. 6B), the transfection with *GLUD2* siRNA in the presence of empty vector (ev) again prevented NH_4_Cl-induced impairment of respiration (Fig. 6D), thus confirming our knockdown results. Intrigued by these results, pointing to a critical role of GDH2, and by the metabolomics data showing a long-term increase in numerous amino acids, we reasoned that, in particular, glutamate levels might be altered in a rapid manner by ammonia. Astrocytoma cells indeed showed a gross increase in glutamate levels immediately after exposure to ammonia (Fig. 6G). This was not due to altered protein levels of GDH2 or its regulator SIRT4 (Fig. S9A,B), nor due to altered specific GDH activity induced by the addition of ammonia (Fig. S10A). Taken together, these results provide strong evidence that GDH2 is a critical factor in the rapid impairment of mitochondrial respiration caused by hyperammonemia.

### Inhibition of GDH-dependent reductive amination of α-ketoglutarate by supplementation with glutamate or glutamine, or by SIRT4 overexpression, improves mitochondrial respiration under hyperammonemia

To further test the role of GDH in ammonia-induced toxicity of mitochondrial respiration, we aimed to test whether the detrimental effects of ammonia can be reduced using different amino acids. Pretreatment with 10 mM glutamine or glutamate directly before treatment with 5 mM NH_4_Cl for 1 h both led to reduction of the ammonia-mediated decrease in OCR (Fig. 6H,I). This is consistent with the rapid ability to produce α-ketoglutarate directly from glutamate or indirectly by producing glutamate from glutamine by mitochondrial glutaminase. The rapid incorporation of ammonia-derived nitrogen in glutamate (Fig. 5A-F), the increase in total glutamate levels upon ammonia treatment (Fig. 6G) and our siRNA experiments (Fig. 6A-D) strongly suggest a crucial role of GDH2 in mediating glutamate- and glutamine-dependent restoration of mitochondrial respiration. To corroborate this further, we applied a metabolic tracing experiment using ^13^C-glutamine, allowing us to study glutamine-dependent anaplerosis of TCA intermediates, or glutaminolysis, under norm- and hyperammonemic conditions. Human astrocytoma cells were treated with a medium containing 2 mM or 10 mM fully ^13^C-isotope-labeled glutamine with or without 5 mM NH_4_Cl for 40 min. After ammonia treatment at normal (2 mM) glutamine concentrations, the total levels of α-ketoglutarate, fumarate and malate were significantly decreased, confirming an ammonia-induced depletion of TCA cycle intermediates within 40 min (Fig. S11, Table S5). This depletion was significantly inhibited by high concentrations (10 mM) of glutamine (Fig. S11), explaining why glutamine can restore mitochondrial respiration (Fig. 6I). The anaplerotic incorporation of ^13^C derived from glutamine into TCA cycle intermediates such as α-ketoglutarate (M+5), malate (M+4) and fumarate (M+4) was significantly inhibited by hyperammonemia only when glutamine concentration were normal, but not when glutamine concentrations were high in the medium (Fig. S11). This confirms that, under hyperammonemia, promoting glutaminolysis is a possible strategy to overcome mitochondrial toxicity of ammonia.

GDH is known to be inhibited by ADP ribosylation catalyzed by the mitochondrial sirtuin SIRT4 (Haigis et al., 2006). We employed HeLa-SIRT4-eGFP cells, a stable cell line overexpressing SIRT4, and the corresponding control cells (HeLa-eGFP) to test whether inhibition of mitochondrial GDH by SIRT4 can also restore mitochondrial respiration and/or mitochondrial morphology upon hyperammonemia. First, we confirmed that SIRT4-GFP overexpression (Fig. S7) caused a significant reduction in specific GDH activity compared to control cells expressing only GFP (Fig. 6E). Mitochondrial morphology and response to ammonia were not affected by expression of SIRT4-GFP (Fig. S8). Also, the spare respiratory capacities of untreated HeLa-SIRT4-eGFP and HeLa-eGFP cells were not affected. The fact that mitochondrial morphology was not restored by SIRT4 overexpression supports the view that ammonia exerts additional, detrimental effects that are independent of GDH. In line with this, ammonia results in a reduction in the mitochondrial membrane potential and in increased ROS formation, two parameters known to cause mitochondrial fragmentation (Görg et al., 2013; Duvezin-Caubet et al., 2006; Lu et al., 2019; Legros et al., 2002). However, after 1 h ammonia treatment, respiration dropped significantly in HeLa-eGFP cells but not in cells overexpressing SIRT4 (Fig. 6F), demonstrating that SIRT4 overexpression efficiently prevented ammonia-induced detrimental effects on mitochondrial respiration.

**Fig. 7.**
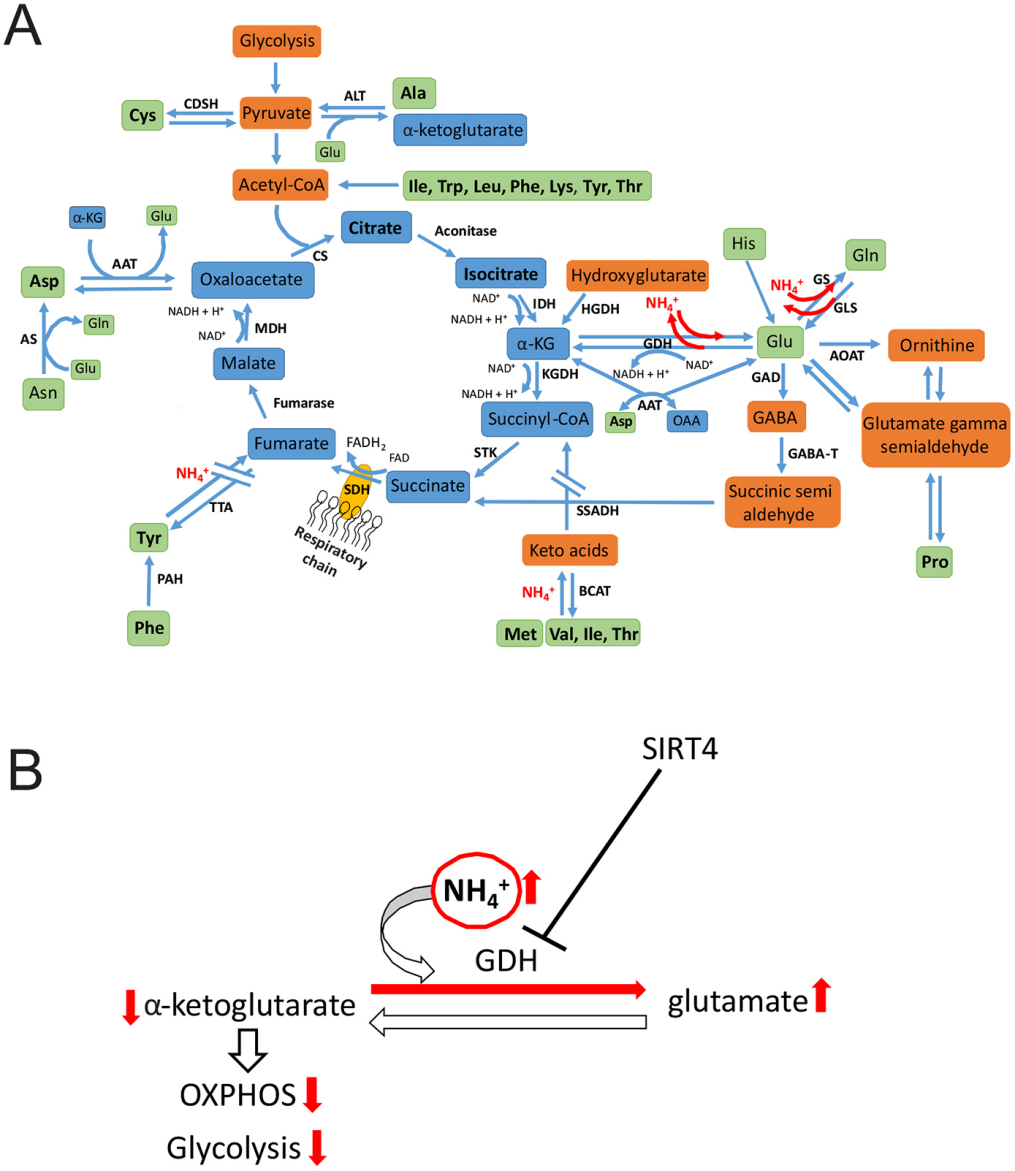
**Proposed mechanism of the influence of ammonia on mitochondrial metabolism.** (A) TCA cycle and anaplerotic reactions including the role of ammonia. Amino acids and TCA cycle intermediates increased in steady-state metabolites are depicted in bold. (B) Proposed mechanism on the role of GDH in ammonia-induced toxicity. α-KG, α-ketoglutarate; AAT, aspartate aminotransferase; ALT, alanine aminotransferase; AOAT, acetyl ornithine aminotransferase; AS, asparaginase; BCAT, branched-chain amino acid transferase; CDSH, cysteine desulfhydrase; CS, citrate synthase; GABA-T, GABA-transaminase; GAD, glutamate decarboxylase; GDH, glutamate dehydrogenase; GLS, glutaminase; GS, glutamine synthetase; HGDH, hydroxyglutarate dehydrogenase; IDH, isocitrate dehydrogenase; KGDH, α-ketoglutarate dehydrogenase; MDH, malate dehydrogenase; OAA, oxaloacetate; OXPHOS, oxidative phosphorylation; PAH, phenylalanine hydroxylase; SDH, succinate dehydrogenase; SSADH, succinate semialdehyde dehydrogenase; STK, succinate thiokinase; TTA, tyrosine transaminase.

Overall, several lines of evidence indicate an essential role of mitochondrial GDH2 in the rapid impairment of mitochondrial respiration under hyperammonemia. Mechanistically, we propose that GDH2 is required for ammonia-induced depletion of α-ketoglutarate by reductive amination to glutamate, and that promoting anaplerosis of the TCA cycle via glutaminolysis can overcome the detrimental effects of ammonia on mitochondrial respiration (Fig. 7A,B).

## DISCUSSION

HE is a common complication occurring upon severe liver dysfunction. Although it is largely accepted that hyperammonemia-induced impairment of astrocytes plays a major role in mediating neurological disturbances (Görg et al., 2018; Norenberg, 1987), the underlying molecular mechanisms are unknown and numerous models have been proposed (Cash et al., 2010; Cordoba, 2014; Ott and Vilstrup, 2014). One particular aspect that is controversially discussed is the role of altered brain energy metabolism caused by hyperammonemia. Alterations in the CSF metabolome of HE patients (Weiss et al., 2016), differences in respiratory chain enzyme activities in rat HE models (Boer et al., 2009; Dhanda et al., 2017), and variable results on altered levels/activities of TCA cycle metabolites and enzymes have been reported (Rama Rao and Norenberg, 2012). We noticed that the large majority of studies investigating acute or chronic *in vitro* and *in vivo* HE models address the toxic effects of hyperammonemia after 1 day, a few days or even weeks (Görg et al., 2015; Hazell and Norenberg, 1998; Oenarto et al., 2016). Little is known about the immediate effect of ammonia on brain energy metabolism. Here, we provide several lines of evidence that high ammonia levels lead to a rapid metabolic reprogramming of astrocytes, in particular via GDH-dependent impairment of the TCA cycle, raising the possibility that this represents an early event in the pathogenesis of HE.

The following arguments strongly support this view: (1) our data show that ammonia introduces rapid mitochondrial fragmentation in two cell types, human astrocytoma cells and primary rat astrocytes; (2) in primary cells, the effect was more rapidly observed, which could be attributed to metabolic differences in tumor cells (Hsu and Sabatini, 2008); (3) hyperammonemia resulted in an instantaneous impairment of mitochondrial respiration in both cell types; (4) the effect was not mediated by possible changes in pH as a pH mimetic did not impair respiration; (5) glycolysis was markedly hampered during hyperammonemia as a consequence of TCA cycle inhibition; (6) metabolomic analyses revealed a delayed increase of several amino acids, including those that can directly or indirectly engage in anaplerotic reactions feeding the TCA cycle; (7) glucose levels were increased, consistent with the observation that glycolysis is inhibited rapidly by ammonia; (8) isotope labeling of ammonia and metabolic ^15^N tracing showed that ammonia is rapidly fixed in glutamate (and metabolites derived from glutamate), which prompted us to investigate the role of the GDH that catalyzes the reductive amination of α-ketoglutarate and ammonia to glutamate; (9) ammonia-induced inhibition of mitochondrial respiration is strongly dependent on the mitochondrial GDH2, suggesting that removal of ammonia may occur via GDH2; (10) GDH2 inhibition by overexpression of the mitochondrial sirtuin SIRT4 has the same effect as GDH2 knockdown; (11) GDH2 overexpression resulted in a sensitization of astrocytes to ammonia; (12) glutamate levels increased instantaneously after administration of ammonia and a decrease in TCA intermediates was observed 40 min after ammonia addition; (13) glutamate/glutamine addition rescued respiratory effects, suggesting that promoting anaplerosis of the TCA cycle via GDH2 can reduce ammonia-induced toxicity; and (14) this was confirmed by ^13^C-metabolic tracing of glutamine under norm- and hyperammonemia, showing that promoting glutaminolysis under hyperammonemia was able to restore TCA cycle intermediates. Taken together, we have multiple lines of evidence that a critical entry point of excessive ammonia is the reductive amination of α-ketoglutarate by GDH2, causing impairment of the TCA cycle and thus of respiration as well as glycolysis. GDH2 is known to play an important part specifically in astrocytes, yet its role under hyperammonemia is not well understood. It was reported that *GLUD2* expression in astrocytes increases the capacity for uptake and oxidative metabolism of glutamate, particularly during increased workload and aglycemia, implying that GDH2 is an important mediator allowing the replenishment of the TCA cycle by glutamate (Nissen et al., 2018). This is in line with our data showing that glutamate can indeed ameliorate the effects of hyperammonemia. A recent study showed that metabolic differences between transgenic mice carrying the human gene *GLUD2* and control mice during postnatal brain development center on metabolic pathways surrounding the TCA cycle (Li et al., 2016). These results support the importance of the GDH2-dependent modulation of energy metabolism in the brain.

In contrast to the prevailing view for norm conditions, our data show that, under hyperammonemic conditions, the net conversion of α-ketoglutarate occurs primarily towards glutamate and not vice versa. To our knowledge, this direction of the GDH reaction has not been considered to be relevant for the pathogenesis of HE so far. This does not contradict the role of GDH as an important enzyme for anaplerosis, which is undisputed under normal conditions. An ammonia-detoxifying role of GDH has been shown in the liver (Williamson et al., 1967) and was proposed based on a mathematical model employed to investigate the mechanism of ammonia detoxification (Ghallab et al., 2016). Consistent with this, injection of GDH and α-ketoglutarate with cofactors into mice reduced ammonia blood levels to normal levels within 15 min (Ghallab et al., 2016). Additionally, a rapid decrease in α-ketoglutarate concentration in rat liver was reported after injection of NH_4_Cl solution (Williamson et al., 1967). To our knowledge, the role of GDH-dependent ammonia detoxification in tissues other than the liver has not been addressed so far. Thus, our data now show that GDH2 in human astrocytes, on one hand, helps to remove the ammonia load, but, on the other hand, impairs the TCA cycle. GDH2-mediated removal of ammonia is not only beneficial, as commonly expected, but also detrimental to astrocytes. As a result of impairing the TCA cycle, we can well explain why glycolysis, as well as OXPHOS, is rapidly inhibited in hyperammonemia. We see a clear concentration dependency of ammonia-induced effects on respiration. This is in accordance with most patient data showing a correlation between blood ammonia levels and severity of disease (Ong et al., 2003).

It is interesting to note that humans and apes (hominoids), but not other mammals, have two genes encoding GDH1 and GDH2, namely *GLUD1* and *GLUD2*, respectively. GDH1 is found in all mammals in the cytosol and mitochondria and is widely expressed in all tissues. *GLUD2* encodes a mitochondrial form of GDH that is expressed solely in the testes, epithelial kidney cells and astrocytes of hominoids. It is proposed that *GLUD2* appeared in evolution after retroposition of the *GLUD1* gene, probably in an ape ancestor less than 23 million years ago (Burki and Kaessmann, 2004). The mature forms of human GDH1 and GDH2 are highly homologous, with a sequence similarity of ∼97%. Under physiological conditions, the GDH reaction mainly catalyzes the oxidative deamination to form α-ketoglutarate (Adeva et al., 2012). GDH is regulated by SIRT4, a mitochondrial enzyme that uses NAD^+^ to ADP-ribosylate GDH and that inhibits GDH activity (Haigis et al., 2006). Here, we show that overexpression of SIRT4 can phenocopy the effect of GDH2 knockdown, corroborating our model.

One strong argument allowing us to conclude that ammonia is utilized in the GDH reaction towards glutamate was obtained from ^15^N label incorporation mainly in glutamate, aspartate and proline, as well as to a lower extent in leucine, isoleucine, valine, alanine and histidine after administration of ^15^NH_4_Cl. Glutamate, aspartate, proline and the BCAAs are directly associated with GDH downstream reactions or acquire the ^15^N label through secondary reactions. It is interesting to note that breast cancer cells have also been shown to fix ammonia via the GDH reaction; Spinelli et al. (2017) reported that, in breast cancer cell lines, ammonia is primarily assimilated through reductive amination catalyzed by GDH, and the high accumulation of labeled nitrogen in glutamate, proline, aspartate and alanine, among others, is strongly reminiscent of our data in astrocytes.

Other studies support the critical role of the TCA cycle in HE. Weiss et al. (2016) examined metabolomics to highlight the dysfunctions of metabolic pathways in CSF samples of HE patients. This revealed an accumulation of acetylated compounds, which also points towards a defect in the TCA cycle. Additionally, the increased metabolites are involved in ammonia, amino acid and energy metabolism; for example, glutamate, glutamine, methionine, phenylalanine and others (Weiss et al., 2016), many of which we also found elevated. Patient data show an association between arterial hyperammonemia and increase in glutamine concentration in the brain (Tofteng et al., 2006), which could result from GDH activity towards glutamate and, subsequently, glutamine. It was further shown that, under normal conditions, GDH is important to sustain the catalytic activity of the TCA cycle in mouse astrocytes by mediating the net formation of TCA intermediates, and that reduced GDH expression induces the usage of alternative substrates such as BCAAs (Nissen et al., 2015).

In contrast, we show that, at high ammonia levels, with increased time the concentration of amino acids such as BCAAs increases. Why is the effect of ammonia on mitochondrial respiration most prevalent at short time points but becomes less pronounced with time? We attribute this to a compensatory mechanism that involves the induction of anaplerotic reactions other than the one catalyzed by GDH. Such a mechanism could be enhanced proteolysis by autophagy or proteasomal degradation of proteins. A recent study reported that autophagic flux is altered in astrocytes in various HE models (Lu et al., 2019). Autophagy was also found to be induced in the substantia nigra of mice with liver damage and subsequent hyperammonemia (Bai et al., 2018). In our study, when ammonia was washed out for only 1 h, the detrimental effect on mitochondrial respiration had not only gone, but mitochondrial respiration had reached levels above control levels, in particular after longer pretreatments with ammonia. This could be due to the fact that accumulated amino acids can rapidly engage in anaplerotic reactions and drive the TCA cycle as soon as ammonia is removed. Further, the detrimental effects of ammonia are rapidly reversible, which was also seen in mitochondrial morphology.

The importance of GDH for mitochondrial respiration is also known from *in vivo* studies. In Cns–*Glud1^−/−^* mice (GDH1 knockout in synaptosomes), the basal respiration in brain mitochondria in the presence of glutamate and malate was significantly reduced (Hohnholt et al., 2017). In GDH1 knockout neurons without stimulation of respiration (e.g. by FCCP treatment), there is no effect of GDH1 knockout on respiration, whereas upon stimulation by FCCP, the cells do not respond with an increase in respiration (Hohnholt et al., 2017). This is in line with our results showing that hyperammonemia does not grossly affect basal mitochondrial respiration but rather only affects it when respiratory activity is induced.

Our *in vitro* findings have identified two novel factors, GDH2 and SIRT4, and demonstrate a crucial role of the TCA cycle in the pathogenesis of HE. This may help to develop new treatment options in HE, e.g. supplementation of certain amino acids promoting anaplerosis, such as glutamate or glutamine. Targeting sirtuins, in particular SIRT1-3 and SIRT5, is already being studied intensively in cancer research. SIRT4 is regulated by miR15a/b (Lang et al., 2016), but a specific compound inhibiting SIRT4 activity is not known so far. Targeting SIRT4 could present another promising future therapeutic target for treating patients suffering from HE. Further studies, especially on the potential role of SIRT4 and GDH regulation, are needed for the development of improved treatment strategies for HE symptoms.

## MATERIALS AND METHODS

### Cell lines

Human astrocytoma cells (MOG-G-CCM) from European Collection of Authenticated Cell Cultures (ECACC; Public Health England, Salisbury, UK) were established from an anaplastic astrocytoma of human adult brain. Cells were grown in Dulbecco's modified eagle medium (DMEM) with 1 g/l glucose (Sigma-Aldrich, Taufkirchen, Germany), with 10% fetal calf serum (PAN-Biotech, Aidenbach, Germany), 2 mM GlutaMax (Thermo Fisher Scientific, Carlsbad, CA, USA), 2 mM sodium pyruvate (Thermo Fisher Scientific) and penicillin/streptomycin (Merck Millipore, Burlington, MA, USA) at 37°C and 5% humidified CO_2_. Primary rat astrocytes were prepared from the cerebral hemispheres of newborn Wistar rats and grown under the same conditions. The care and use of experimental animals complied with all relevant local animal welfare laws, guidelines and policies. SIRT4-eGFP-overexpressing HeLa cells and respective eGFP control cells were established as described here and in the Supplementary Materials and Methods and grown under the same conditions in the presence of 2 µg/ml puromycin (Thermo Fisher Scientific).

### Cellular metabolism analysis

A Mito Stress Test Kit and a Glycolysis Stress Test Kit (Agilent Technologies, Santa Clara, CA, USA) were applied according to the manufacturer's instructions using a Seahorse XFe96 Extracellular Flux Analyzer (Agilent Technologies). FCCP concentration and cell density were titrated/determined for each cell type prior to the experiments. Treatment with NH_4_Cl (VWR, Radnor, PA, USA) or CH_3_NH_3_Cl (Merck Millipore) at the given molarity was performed for 1, 4, 6, 24 and 48 h, or immediately prior to analysis (0 h). After measurement, cell numbers were quantified by absorption spectrometry (excitation, 361 nm; emission, 486 nm) using Hoechst 33342 (Thermo Fisher Scientific) staining using a plate reader (Tecan Infinite 200 PRO, Switzerland) and normalized to cell number. Basal respiration is defined as the respiration before the first injection by the Seahorse system.

Maximal respiration was defined as the OCR after FCCP injection (maximal OCR) – the OCR after blocking mitochondrial respiration (non-mitochondrial OCR). Spare respiratory capacity was defined as the maximal OCR – basal respiration. Spare respiratory capacity (%) was defined as maximal respiration/basal respiration×100. Maximal respiration and spare respiratory capacity are challenged states of respiration. Glycolysis was the ECAR: the maximum rate measurement before oligomycin injection – the last rate of measurement before glucose injection. Glycolytic capacity was the maximum rate measurement after injection of oligomycin – the last rate measurement before glucose injection.

### Plasmids and transfection of cell lines

To visualize mitochondria, the construct pEGFP-Mito (Clontech Laboratories, Mountain View, CA, USA) was used. Transfection was performed using Effectene Transfection Reagent (Qiagen, Hilden, Germany) according to the manufacturer's instructions 24 h before imaging. Knockdown of GDH2 using siRNAs (#NM_012084: 5′-CUAACCUCUUCACGUGUAA-3′ and 5′-UUACACGUGAAGAGGUUAG-3′, Sigma-Aldrich) and transfection of *GLUD2* plasmid/empty vector controls were performed using Lipofectamine RNAiMAX (Thermo Fisher Scientific) for 48 h in Seahorse plates, according to the manufacturer's instructions using the reverse transfection protocol. Human *GLUD2* was cloned into pcDNA3.1(+) (clone ID OHu18663, obtained from GenScript USA, Piscataway, NJ, USA). Control vector was pcDNA3.1(+) (Invitrogen, Thermo Fisher Scientific). The DNA sequence in the GLUD2 overexpression plasmid was on purpose not targeted by the siRNA by introducing suitable silent mutations.

### Microscopy

Human astrocytoma cells were seeded in 3 cm glass bottom dishes (MatTek Corporation, Ashland, MA, USA), transfected with pEGFP-Mito and treated with 5 mM NH_4_Cl for 1, 4, 6, 24, 48 or 72 h. For Fig. S8, HeLa-(SIRT4)-eGFP cells were seeded in the same dishes described above and treated with 5 mM NH_4_Cl for 24 h or 48 h. Imaging was performed with a Zeiss Axiovert Observer D1 microscope with a 63×/1.4 NA oil objective (Filter: excitation, 450-490 nm; emission, 500-550 nm) (Zeiss, Oberkochen, Germany) and AxioVision Software. Twenty images per dish were taken and cells were categorized and quantitatively scored according to the degree of fragmentation; (tubular, intermediate, fragmented: cells with >90%, 90-10%, <10% of total mitochondrial signal with tubular morphology, respectively; Fig. 1A). For reversibility of the mitochondrial phenotype, primary rat astrocytes transfected with pEGFP-Mito were grown in the presence of 5 mM NH_4_Cl for 72 h and analyzed for mitochondrial fragmentation. Medium was exchanged and cells were analyzed for mitochondrial morphology 24 h, 48 h and 72 h after the removal of NH_4_Cl. To determine mitochondrial morphology changes in primary rat astrocytes, mitochondria were visualized by immunostaining against Tom20 (primary antibody: sc-11415, Santa Cruz Biotechnology, Dallas, TX, USA; secondary antibody: Alexa Fluor 488, #A27034, Thermo Fisher Scientific). To determine SIRT4 localization in HeLa-(SIRT4)-eGFP cells, the nucleus was stained with DAPI (Sigma-Aldrich), and for mitochondrial visualization immunostaining was performed against Tom20 (primary antibody: see above; secondary antibody: Alexa Fluor 546 #A11035, Invitrogen, Thermo Fisher Scientific). Analysis was performed as described above. Images were taken using a Spinning Disk Confocal microscope (PerkinElmer, Waltham, MA, USA) using the 405 nm, 488 nm and 561 nm lasers, 60×/1.4 NA oil objective and Volocity software. Images were processed with Fiji (Schindelin et al., 2012).

### Metabolite analysis

Cells were grown in 175-cm^2^ flasks (Greiner Bio-One, Kremsmünster, Austria). For metabolite screening and ^15^N-metabolic tracing, cells were treated with 5 mM NH_4_Cl for 48, 24, 6, 4, 2 or 1 h or harvested immediately after treatment (0 h). For ^13^C-metabolic tracing, cells were treated with medium containing 2 mM or 10 mM [U-^13^C]-glutamine (#605166, Millipore Sigma, Taufkirchen, Germany) and 5 mM NH_4_Cl or water as control for 40 min. Cells were washed with PBS, trypsinized and resuspended in DMEM for cell counting. For metabolite screening and ^15^N-metabolic tracing, 1×10^6^ cells were pelleted, and for ^13^C-metabolic tracing, 3×10^6^ cells were pelleted. Pellets were washed twice with ice-cold PBS and resuspended in a pre-cooled (−20°C) 1:2.5:1 mixture of H_2_O: methanol:chloroform (both VWR, Radnor, PA, USA), mixed at 4°C for 10 min, and centrifuged at 9300 ***g*** for 5 min at 4°C. The supernatant was subject to metabolite profiling. Polar metabolites were analyzed by gas chromatography coupled to a time-of-flight (TOF) mass spectrometer (GC-QTOF) (7200 GC-QTOF, Agilent Technologies) as published (Fiehn and Kind, 2007). For relative quantification, peak areas of the compounds were normalized to the internal standard ribitol (Sigma-Aldrich) added to the extraction buffer. To follow accumulation of ^15^N label, cells were treated with ^15^NH_4_Cl as described above. Metabolites were analyzed by liquid chromatography coupled to a TOF mass spectrometer (LC-QTOF) (1290 UHPLC 6530 QTOF, Agilent Technologies) according to Gu et al. (2007). Relative ^15^N label enrichment was calculated after accounting for natural isotopic distribution. Peak integration and analysis were performed using the Agilent Mass Hunter Workstation B07 (Agilent Technologies). Further details are provided in Tables S1-S3. For ion chromatography-mass spectrometry (IC-MS) analysis, a combination of a Dionex ICS-6000 HPIC and a Thermo Fisher Scientific Q Exactive Plus mass spectrometer was used following the method described by Schwaiger et al. (2017) with slight modifications. In brief, the dried sample was reconstituted in 100 µl deionized water, of which 5 µl was injected via a Dionex AS-AP autosampler. For the anion exchange chromatography, a Dionex IonPac AS11-HC column (2 mm×250 mm, 4 μm particle size, Thermo Fisher Scientific) equipped with a Dionex IonPac AG11-HC guard column (2 mm×50 mm, 4 μm, Thermo Fisher Scientific) was used, and the mobile phase was generated using an eluent generator with a potassium hydroxide cartridge. The mass spectrometer operated in negative mode with a combination of full mass scan and a data-dependent Top5 MS^2^ (ddMS^2^) experiment with a resolution of 140,000 and 17,500, respectively. Data analysis was conducted using a Compound Discoverer (version 3.1, Thermo Fisher Scientific). For data analysis, the standard workflow for stable isotope labeling from the Compound Discoverer was chosen and the default settings were used: 5 ppm mass tolerance, 30% intensity tolerance and 0.1% intensity threshold for isotope pattern matching. As an additional level of validation, an in-house database for retention times and MS^2^ spectra was created using mzVault (Thermo Fisher Scientific) and implemented in the annotation workflow.

### GDH activity kit

To determine changes in the activity of GDH under various conditions, a colorimetric Glutamate Dehydrogenase (GDH) Activity Assay Kit (MAK099, Sigma-Aldrich) was employed according to the manufacturer's instructions. In brief, cells were treated with the respective condition [5 mM NH_4_Cl for 1 h and 24 h; *GLUD2* knockdown and overexpression as described above; HeLa-(SIRT4)-eGFP cells untreated] and 1×10^6^ cells per sample were used for the assay. Cells were homogenized in ice-cold assay buffer and used as described in the instructions. From colorimetric measurements, the amount of NADH generated in nmol and the GDH activity in mU/ml were determined using a standard curve. Bradford measurement was performed for all samples and results were corrected for protein concentration to obtain specific enzyme activities.

### Statistics

Statistics were performed using GraphPad Prism 7.04 for Windows (GraphPad Software, La Jolla, CA, USA). For multiple comparisons, one-way ANOVA with Dunnett's, Tukey's or Sidak's post hoc test or paired one-way ANOVA with Geisser–Greenhouse correction and Sidak's multiple comparison post hoc test was performed. To compare two groups, Student's *t*-test was applied. To compare one group to a normalized value, one-sample *t*-test was used. Data are shown as mean±s.e.m. or s.d., as indicated in the figure legends.

## Supplementary Material

Supplementary information

## References

[DMM047134C1] Adeva M. M., Souto G., Blanco N. and Donapetry C. (2012). Ammonium metabolism in humans. *Metabolism* 61, 1495-1511. 10.1016/j.metabol.2012.07.007 22921946

[DMM047134C2] Bai G., Rama Rao K. V., Murthy C. R., Panickar K. S., Jayakumar A. R. and Norenberg M. D. (2001). Ammonia induces the mitochondrial permeability transition in primary cultures of rat astrocytes. *J. Neurosci. Res.* 66, 981-991. 10.1002/jnr.10056 11746427

[DMM047134C3] Bai Y., Wang Y. and Yang Y. (2018). Hepatic encephalopathy changes mitochondrial dynamics and autophagy in the substantia nigra. *Metab. Brain Dis.* 33, 1669-1678. 10.1007/s11011-018-0275-6 29998403

[DMM047134C4] Boer L. A., Panatto J. P., Fagundes D. A., Bassani C., Jeremias I. C., Daufenbach J. F., Rezin G. T., Constantino L., Dal-Pizzol F. and Streck E. L. (2009). Inhibition of mitochondrial respiratory chain in the brain of rats after hepatic failure induced by carbon tetrachloride is reversed by antioxidants. *Brain Res. Bull.* 80, 75-78. 10.1016/j.brainresbull.2009.04.009 19406217

[DMM047134C5] Burki F. and Kaessmann H. (2004). Birth and adaptive evolution of a hominoid gene that supports high neurotransmitter flux. *Nat. Genet.* 36, 1061-1063. 10.1038/ng1431 15378063

[DMM047134C6] Cash W. J., McConville P., McDermott E., McCormick P. A., Callender M. E. and McDougall N. I. (2010). Current concepts in the assessment and treatment of Hepatic Encephalopathy. *QJM* 103, 9-16. 10.1093/qjmed/hcp152 19903725

[DMM047134C7] Cordoba J. (2014). Hepatic encephalopathy: from the pathogenesis to the new treatments. *ISRN Hepatology* 2014, 236268 10.1155/2014/236268 27335836 PMC4890879

[DMM047134C8] Dhanda S., Sunkaria A., Halder A. and Sandhir R. (2017). Mitochondrial dysfunctions contribute to energy deficits in rodent model of hepatic encephalopathy. *Metab. Brain Dis.* 33, 209-223. 10.1007/s11011-017-0136-8 29138968

[DMM047134C9] Duvezin-Caubet S., Jagasia R., Wagener J., Hofmann S., Trifunovic A., Hansson A., Chomyn A., Bauer M. F., Attardi G., Larsson N.-G. et al. (2006). Proteolytic processing of OPA1 links mitochondrial dysfunction to alterations in mitochondrial morphology. *J. Biol. Chem.* 281, 37972-37979. 10.1074/jbc.M606059200 17003040

[DMM047134C10] Ferenci P. (2017). Hepatic encephalopathy. *Gastroenterol. Rep.* 5, 138-147. 10.1093/gastro/gox013 PMC5421503 28533911

[DMM047134C11] Ferenci P., Lockwood A., Mullen K., Tarter R., Weissenborn K. and Blei A. T. (2002). Hepatic encephalopathy—definition, nomenclature, diagnosis, and quantification: final report of the working party at the 11th world congresses of gastroenterology, Vienna, 1998. *Hepatology* 35, 716-721. 10.1053/jhep.2002.31250 11870389

[DMM047134C12] Fiehn O. and Kind T. (2007). Metabolite profiling in blood plasma. *Methods Mol. Biol.* 358, 3-17. 10.1007/978-1-59745-244-1_1 17035677

[DMM047134C13] Frank M., Duvezin-Caubet S., Koob S., Occhipinti A., Jagasia R., Petcherski A., Ruonala M. O., Priault M., Salin B. and Reichert A. S. (2012). Mitophagy is triggered by mild oxidative stress in a mitochondrial fission dependent manner. *Biochim. Biophys. Acta* 1823, 2297-2310. 10.1016/j.bbamcr.2012.08.007 22917578

[DMM047134C14] Ghallab A., Cellière G., Henkel S. G., Driesch D., Hoehme S., Hofmann U., Zellmer S., Godoy P., Sachinidis A., Blaszkewicz M. et al. (2016). Model-guided identification of a therapeutic strategy to reduce hyperammonemia in liver diseases. *J. Hepatol.* 64, 860-871. 10.1016/j.jhep.2015.11.018 26639393

[DMM047134C15] Görg B., Qvartskhava N., Keitel V., Bidmon H. J., Selbach O., Schliess F. and Häussinger D. (2008). Ammonia induces RNA oxidation in cultured astrocytes and brain in vivo. *Hepatology* 48, 567-579. 10.1002/hep.22345 18506841

[DMM047134C16] Görg B., Schliess F. and Häussinger D. (2013). Osmotic and oxidative/nitrosative stress in ammonia toxicity and hepatic encephalopathy. *Arch. Biochem. Biophys.* 536, 158-163. 10.1016/j.abb.2013.03.010 23567841

[DMM047134C17] Görg B., Karababa A., Shafigullina A., Bidmon H. J. and Häussinger D. (2015). Ammonia-induced senescence in cultured rat astrocytes and in human cerebral cortex in hepatic encephalopathy. *Glia* 63, 37-50. 10.1002/glia.22731 25092802

[DMM047134C18] Görg B., Karababa A. and Häussinger D. (2018). Hepatic encephalopathy and astrocyte senescence. *J. Clin. Exp. Hepatol.* 8, 294-300. 10.1016/j.jceh.2018.05.003 30302047 PMC6175776

[DMM047134C19] Gu L., Jones A. D. and Last R. L. (2007). LC-MS/MS assay for protein amino acids and metabolically related compounds for large-scale screening of metabolic phenotypes. *Anal. Chem.* 79, 8067-8075. 10.1021/ac070938b 17918906

[DMM047134C20] Haigis M. C., Mostoslavsky R., Haigis K. M., Fahie K., Christodoulou D. C., Murphy Andrew J., Valenzuela D. M., Yancopoulos G. D., Karow M., Blander G. et al. (2006). SIRT4 inhibits glutamate dehydrogenase and opposes the effects of calorie restriction in pancreatic β cells. *Cell* 126, 941-954. 10.1016/j.cell.2006.06.057 16959573

[DMM047134C21] Hazell A. S. and Norenberg M. D. (1998). Ammonia and manganese increase arginine uptake in cultured astrocytes. *Neurochem. Res.* 23, 869-873. 10.1023/A:1022411012512 9572676

[DMM047134C22] Hohnholt M. C., Andersen V. H., Andersen J. V., Christensen S. K., Karaca M., Maechler P. and Waagepetersen H. S. (2017). Glutamate dehydrogenase is essential to sustain neuronal oxidative energy metabolism during stimulation. *J. Cereb. Blood Flow Metab.* 38, 1754-1768. 10.1177/0271678X17714680 28621566 PMC6168903

[DMM047134C23] Hsu P. P. and Sabatini D. M. (2008). Cancer cell metabolism: warburg and beyond. *Cell* 134, 703-707. 10.1016/j.cell.2008.08.021 18775299

[DMM047134C24] Jheng H.-F., Tsai P.-J., Guo S.-M., Kuo L.-H., Chang C.-S., Su I.-J., Chang C.-R. and Tsai Y.-S. (2012). Mitochondrial fission contributes to mitochondrial dysfunction and insulin resistance in skeletal muscle. *Mol. Cell. Biol.* 32, 309-319. 10.1128/MCB.05603-11 22083962 PMC3255771

[DMM047134C25] Katunuma N., Okada M. and Nishii Y. (1966). Regulation of the urea cycle and TCA-cycle by ammonia. *Adv. Enzyme Regul.* 4, 317-336. 10.1016/0065-2571(66)90025-2 4229888

[DMM047134C26] Lang A., Grether-Beck S., Singh M., Kuck F., Jakob S., Kefalas A., Altinoluk-Hambüchen S., Graffmann N., Schneider M., Lindecke A. et al. (2016). MicroRNA-15b regulates mitochondrial ROS production and the senescence-associated secretory phenotype through sirtuin 4/SIRT4. *Aging* 8, 484-505. 10.18632/aging.100905 26959556 PMC4833141

[DMM047134C27] Larsen F. S., Ranek L., Hansen B. A. and Kirkegaard P. (1995). Chronic portosystemic hepatic encephalopathy refractory to medical treatment successfully reversed by liver transplantation. *Transpl. Int.* 8, 246-247. 10.1111/j.1432-2277.1995.tb01513.x 7626189

[DMM047134C28] Legros F., Lombès A., Frachon P. and Rojo M. (2002). Mitochondrial fusion in human cells is efficient, requires the inner membrane potential, and is mediated by mitofusins. *Mol. Biol. Cell* 13, 4343-4354. 10.1091/mbc.e02-06-0330 12475957 PMC138638

[DMM047134C29] Li Q., Guo S., Jiang X., Bryk J., Naumann R., Enard W., Tomita M., Sugimoto M., Khaitovich P. and Pääbo S. (2016). Mice carrying a human GLUD2 gene recapitulate aspects of human transcriptome and metabolome development. *Proc. Natl. Acad. Sci. USA* 113, 5358-5363. 10.1073/pnas.1519261113 27118840 PMC4868425

[DMM047134C30] Lockwood A. H., Finn R. D., Campbell J. A. and Richman T. B. (1980). Factors that affect the uptake of ammonia by the brain: the blood-brain pH gradient. *Brain Res.* 181, 259-266. 10.1016/0006-8993(80)90611-3 7350966

[DMM047134C31] Lockwood A. H., McDonald J. M., Reiman R. E., Gelbard A. S., Laughlin J. S., Duffy T. E. and Plum F. (1979). The dynamics of ammonia metabolism in man. Effects of liver disease and hyperammonemia. *J. Clin. Investig.* 63, 449-460. 10.1172/JCI109322 429564 PMC371973

[DMM047134C32] Lu K., Zimmermann M., Görg B., Bidmon H.-J., Biermann B., Klöcker N. Häussinger D. and Reichert A. S. (2019). Hepatic encephalopathy is linked to alterations of autophagic flux in astrocytes. *EBioMedicine* 48, 539-553. 10.1016/j.ebiom.2019.09.058 31648987 PMC6838440

[DMM047134C33] Martinez-Hernandez A., Bell K. P. and Norenberg M. D. (1977). Glutamine synthetase: glial localization in brain. *Science* 195, 1356-1358. 10.1126/science.14400 14400

[DMM047134C34] Nissen J. D., Pajęcka K., Stridh M. H., Skytt D. M. and Waagepetersen H. S. (2015). Dysfunctional TCA-cycle metabolism in glutamate dehydrogenase deficient astrocytes. *Glia* 63, 2313-2326. 10.1002/glia.22895 26221781

[DMM047134C35] Nissen J. D., Lykke K., Bryk J., Stridh M. H., Zaganas I., Skytt D. M., Schousboe A., Bak L. K., Enard W., Pääbo S. et al. (2018). Expression of the human isoform of glutamate dehydrogenase, hGDH2, augments TCA-cycle capacity and oxidative metabolism of glutamate during glucose deprivation in astrocytes. *Glia* 65, 474-488. 10.1002/glia.23105 28032919

[DMM047134C36] Norenberg M. D. (1987). The role of astrocytes in hepatic encephalopathy. *Neurochem. Pathol.* 6, 13-33. 10.1007/BF02833599 3306480

[DMM047134C37] Norenberg M. D., Rao K. V. R. and Jayakumar A. R. (2005). Mechanisms of ammonia-induced astrocyte swelling. *Metab. Brain Dis.* 20, 303-318. 10.1007/s11011-005-7911-7 16382341

[DMM047134C38] Nunnari J., Marshall W. F., Straight A., Murray A., Sedat J. W. and Walter P. (1997). Mitochondrial transmission during mating in Saccharomyces cerevisiae is determined by mitochondrial fusion and fission and the intramitochondrial segregation of mitochondrial DNA. *Mol. Biol. Cell* 8, 1233-1242. 10.1091/mbc.8.7.1233 9243504 PMC276149

[DMM047134C39] Oenarto J., Karababa A., Castoldi M., Bidmon H. J., Görg B. and Häussinger D. (2016). Ammonia-induced miRNA expression changes in cultured rat astrocytes. *Sci. Rep.* 6, 18493 10.1038/srep18493 26755400 PMC4709596

[DMM047134C40] Ong J. P., Aggarwal A., Krieger D., Easley K. A., Karafa M. T., Van Lente F., Arroliga A. C. and Mullen K. D. (2003). Correlation between ammonia levels and the severity of hepatic encephalopathy. *Am. J. Med.* 114, 188-193. 10.1016/S0002-9343(02)01477-8 12637132

[DMM047134C41] Ott P. and Vilstrup H. (2014). Cerebral effects of ammonia in liver disease: current hypotheses. *Metab. Brain Dis.* 29, 901-911. 10.1007/s11011-014-9494-7 24488230

[DMM047134C42] Patidar K. R. and Bajaj J. S. (2015). Covert and overt hepatic encephalopathy: diagnosis and management. *Clin. Gastroenterol. Hepatol* 13, 2048-2061. 10.1016/j.cgh.2015.06.039 26164219 PMC4618040

[DMM047134C43] Polletta L., Vernucci E., Carnevale I., Arcangeli T., Rotili D., Palmerio S., Steegborn C., Nowak T., Schutkowski M., Pellegrini L. et al. (2015). SIRT5 regulation of ammonia-induced autophagy and mitophagy. *Autophagy* 11, 253-270. 10.1080/15548627.2015.1009778 25700560 PMC4502726

[DMM047134C44] Rama Rao K. V. and Norenberg M. D. (2012). Brain energy metabolism and mitochondrial dysfunction in acute and chronic hepatic encephalopathy. *Neurochem. Int.* 60, 697-706. 10.1016/j.neuint.2011.09.007 21989389 PMC4714837

[DMM047134C45] Scaglione S., Kliethermes S., Cao G., Shoham D., Durazo R., Luke A. and Volk M. L. (2015). The epidemiology of cirrhosis in the united states: a population-based study. *J. Clin. Gastroenterol.* 49, 690-696. 10.1097/MCG.0000000000000208 25291348

[DMM047134C46] Schäfer A. and Reichert A. S. (2009). Emerging roles of mitochondrial membrane dynamics in health and disease. *Biol. Chem.* 390, 707-715. 10.1515/BC.2009.086 19453275

[DMM047134C47] Schindelin J., Arganda-Carreras I., Frise E., Kaynig V., Longair M., Pietzsch T., Preibisch S., Rueden C., Saalfeld S., Schmid B. et al. (2012). Fiji: an open-source platform for biological-image analysis. *Nat. Methods* 9, 676-682. 10.1038/nmeth.2019 22743772 PMC3855844

[DMM047134C48] Schwaiger M., Rampler E., Hermann G., Miklos W., Berger W. and Koellensperger G. (2017). Anion-exchange chromatography coupled to high-resolution mass spectrometry: a powerful tool for merging targeted and non-targeted metabolomics. *Anal. Chem.* 89, 7667-7674. 10.1021/acs.analchem.7b01624 28581703

[DMM047134C49] Sontheimer H. (1995). Review: glial neuronal interactions: a physiological perspective. *Neuroscientist* 1, 328-337. 10.1177/107385849500100605

[DMM047134C50] Spinelli J. B., Yoon H., Ringel A. E., Jeanfavre S., Clish C. B. and Haigis M. C. (2017). Metabolic recycling of ammonia via glutamate dehydrogenase supports breast cancer biomass. *Science* 358, 941-946. 10.1126/science.aam9305 29025995 PMC5748897

[DMM047134C51] Stewart V. C., Sharpe M. A., Clark J. B. and Heales S. J. (2000). Astrocyte-derived nitric oxide causes both reversible and irreversible damage to the neuronal mitochondrial respiratory chain. *J. Neurochem.* 75, 694-700. 10.1046/j.1471-4159.2000.0750694.x 10899944

[DMM047134C52] Swain M., Butterworth R. F. and Blei A. T. (1992). Ammonia and related amino acids in the pathogenesis of brain edema in acute ischemic liver failure in rats. *Hepatology* 15, 449-453. 10.1002/hep.1840150316 1544626

[DMM047134C53] Tofteng F., Hauerberg J., Hansen B. A., Pedersen C. B., Jørgensen L. and Larsen F. S. (2006). Persistent arterial hyperammonemia increases the concentration of glutamine and alanine in the brain and correlates with intracranial pressure in patients with fulminant hepatic failure. *J. Cereb. Blood Flow Metab.* 26, 21-27. 10.1038/sj.jcbfm.9600168 15959460

[DMM047134C54] Warskulat U., Görg B., Bidmon H. J., Muller H. W., Schliess F. and Häussinger D. (2002). Ammonia-induced heme oxygenase-1 expression in cultured rat astrocytes and rat brain in vivo. *Glia* 40, 324-336. 10.1002/glia.10128 12420312

[DMM047134C55] Weber T. A., Koob S., Heide H., Wittig I., Head B., Van Der Bliek A., Brandt U., Mittelbronn M. and Reichert A. S. (2013). APOOL is a cardiolipin-binding constituent of the Mitofilin/MINOS protein complex determining cristae morphology in mammalian mitochondria. *PLoS ONE* 8, e63683 10.1371/journal.pone.0063683 23704930 PMC3660581

[DMM047134C56] Weiss N., Barbier Saint Hilaire P., Colsch B., Isnard F., Attala S., Schaefer A., Amador M. M., Rudler M., Lamari F., Sedel F. et al. (2016). Cerebrospinal fluid metabolomics highlights dysregulation of energy metabolism in overt hepatic encephalopathy. *J. Hepatol.* 65, 1120-1130. 10.1016/j.jhep.2016.07.046 27520878

[DMM047134C57] Williamson D. H., Lund P. and Krebs H. A. (1967). The redox state of free nicotinamide-adenine dinucleotide in the cytoplasm and mitochondria of rat liver. *Biochem. J.* 103, 514-527. 10.1042/bj1030514 4291787 PMC1270436

[DMM047134C58] Zalc B. (1994). Astrocytes. *Pharmacol. Funct. J. Neurochem.* 63, 1186 10.1046/j.1471-4159.1994.63031186.x

[DMM047134C59] Zwingmann C., Chatauret N., Leibfritz D. and Butterworth R. F. (2003). Selective increase of brain lactate synthesis in experimental acute liver failure: results of a [^1^H-^13^C] nuclear magnetic resonance study. *Hepatology* 37, 420-428. 10.1053/jhep.2003.50052 12540793

