# Supplementary material for: Ammonia inhibits energy metabolism in astrocytes in a rapid and glutamate dehydrogenase 2-dependent manner
Source: Dis Model Mech. 2020 Nov 4;13(10):dmm047134. doi: 10.1242/dmm.047134 (PMC7657470; doi:10.1242/dmm.047134)
Supplement: Supplementary information [file dmm-13-047134-s1.pdf]

Supplementary Material and Methods

qPCR for *GLUD* knock-down validation

Human astrocytoma cells were seeded and transfected as described before. RNA extraction with All Prep RNA/Protein Kit (Qiagen, Hilden, Germany) according to the manufacturer's instructions. RNA was reverse transcribed to cDNA with QuantiNova Reverse Transcription Kit (Qiagen, Hilden, Germany) according to the manufacturer's instructions. 10 ng cDNA was used for qPCR with Rotorgene 6000 system (Corbett Research, now Qiagen, Hilden, Germany) with the QuantiNova SYBR Green PCR Kit (Qiagen, Hilden, Germany) according to the manufacturer's instructions. Analysis was performed using Rotor-Gene Q Series Software (Qiagen, Hilden, Germany) employing the ΔΔCt method. HPRT1 was used as housekeeping gene. qPCR primer: GLUD2 forward: 5'-cggcagagttccaagacagt-3'; GLUD2 reverse: 5'-gaacgctccattgtgtatgc-3'; HPRT1 forward: 5'-cctggcgtcgtgattagtg-3'; HPRT1 reverse: 5'-tgaggaataaacaccctttcca-3'.

Construction of HeLa-SIRT4-eGFP cells

The cDNAs for eGFP and the human SIRT4-eGFP fusion protein were generated by PCR using pEGFP-N1 and pcDNA3.1-SIRT4-eGFP [1] as templates, respectively, and subsequently cloned via *Nhe*I and *Xho*I restriction sites into puc2CL12IPwo derived from plasmids generated earlier [2, 3]. Constructs were verified by Sanger DNA sequencing. HEK293T cells were transfected as described [2, 3] using polyethylenimine transfection reagent (Sigma-Aldrich, Taufkirchen, Germany) with HIV1 helper plasmid (pCD/NL-BH) [4], envelope vector (pczVSV-G) [5], and the newly generated plasmids puc2CL12eGFPIPwo or puc2CL12SIRT4-eGFPIPwo (both containing an IRES-PuroR cassette). Viral supernatants were harvested 48 h after transfection, filtered through 0.45 µm filters (Sartorius AG, Göttingen, Germany), and used to transduce HeLa cells. Selection with 2 µg/ml puromycin (InvivoGen, San Diego CA, USA) was started 96 h after transduction and eGFP positivity was tracked by flow cytometry (BD FACSCanto II, BD Biosciences, Franklin Lakes NJ, USA) in the FITC-A channel using non-transduced HeLa cells as negative control.

Western Blot

Cells were treated as described previously and harvested with RIPA-buffer (Tris-HCl, NaCl, Triton X-100, sodium deoxycholate, SDS, EDTA, pH 7.4) with Complete protease inhibitor (Roche Diagnostics, Basel, Switzerland) and protein concentration was measured using a Bradford assay (Sigma-Aldrich, Taufkirchen, Germany). Proteins were separated with SDS-PAGE (12 % for GDH2 and SIRT4-eGFP, 10 % for SIRT4, 15 % for UQCRC2, COX8A and NDUFB4), blotted on a nitrocellulose membrane (Amersham, VWR, Radnor PA, USA), and blocked for 1 h with 5% fat-free milk powder solution (Carl Roth GmbH & Co. KG, Karlsruhe, Germany) in TBS. In case of SIRT4 membrane was blocked in 3% BSA (Sigma-Aldrich, Taufkirchen, Germany) solution in TBS-T. Primary antibody against GDH2 (Santa Cruz Biotechnology, Dallas TX, USA, sc-293459) was used at a 1:250 dilution over night; HSP60 antibody (Sigma-Aldrich, Taufkirchen, Germany, SAB4501464) as loading control was used at a 1:10,000 dilution for 1 h at RT. SIRT4 antibody (Proteintech, Rosemont IL, USA, 66543-1-Ig) was used 1:20,000 in TBS-T over night; UQCRC2 (Abcam, Cambridge, UK, ab14745) and COX8A (Thermo Fisher Scientific, Carlsbad CA, USA, PA567695) 1:1000 in 5% fat-free milk powder solution and NDUFB4 (Abcam, Cambridge, UK, ab110243) 1:2000 in 5% milk solution overnight. Loading controls GAPDH (Sigma-Aldrich, Taufkirchen, Germany, G8795) and Tubulin (Cell Signalling Technology, Danvers MA, USA, 2128S) were detected with 1:2000 antibody dilution in 5% milk solution overnight. Respective secondary antibody was decorated in 5% milk solution 1:10,000 at room temperature for 1 h, for SIRT4 antibody was diluted in TBS-T. Blots were developed using Signal Fire ECL Reagent (Cell Signaling Technology, Danvers MA, USA) and visualized using the Fusion SL Gel Documentation System (PEQLAB, Germany).

References

[1] Lang A, Anand R, Altinoluk-Hambüchen S, Ezzahoini H, Stefanski A, Iram A, et al. SIRT4 interacts with OPA1 and regulates mitochondrial quality control and mitophagy. Aging. 2017;9:2163-89.
[2] Roellecke K, Virts EL, Einholz R, Edson KZ, Altvater B, Rossig C, et al. Optimized human CYP4B1 in combination with the alkylator prodrug 4-ipomeanol serves as a novel suicide gene system for adoptive T-cell therapies. Gene therapy. 2016;23:615-26.
[3] Wiek C, Schmidt EM, Roellecke K, Freund M, Nakano M, Kelly EJ, et al. Identification of Amino Acid Determinants in CYP4B1 for Optimal Catalytic Processing of 4-Ipomeanol. The Biochemical journal. 2015;465:103-14.
[4] Zhang X-Y, Russa VFL, Reiser J. Transduction of Bone-Marrow-Derived Mesenchymal Stem Cells by Using Lentivirus Vectors Pseudotyped with Modified RD114 Envelope Glycoproteins. Journal of virology. 2004;78:1219-29.
[5] Pietschmann T, Heinkelein M, Heldmann M, Zentgraf H, Rethwilm A, Lindemann D. Foamy Virus Capsids Require the Cognate Envelope Protein for Particle Export. Journal of virology. 1999;73:2613-21.

Supplemental Tables

Table S1. Raw data for targeted metabolite abundances measured via GC-MS from human astrocytoma cells treated with 5mM NH_4Cl for 1 to 48 hours or not (control).

	Alanine	Valine	Leucine	Isoleucine	Proline	Glycine	Succinic acid	Fumaric acid	Serine	Threonine	Malic acid	Methionine	Aspartic acid	Cysteine
control_1	123.0282066	18.9902447	15.0933263	10.2495073	1.08184889	62.7542946			41.8221387	47.3404029	21.6773031	5.7252943	108.2855737	1.35899103
control_2	82.97457134	16.3380233	21.0935423	14.0284994	5.6429083	9.44571045	4.946868274	10.14652495	27.2109555	33.4481314	5.97195766	6.32663334	59.74423847	2.57441821
control_3	142.9821127	26.204598	31.4617314	19.5112202	10.2114386	19.7211262	4.967354988	10.63390673	21.1035735	41.176682	8.39829423	6.16662224	85.86745617	0.75713076
control_4	10.63989651	1.92692013	2.35237755	1.54077365	1.26079184	0.64434518	1.059071043	1.532604537	1.82952716	2.46674063	0.84682394	0.43191033	7.243406381	0.20234494
control_5														
control_6														
1 h_1	50.41471022	15.1504547	10.5893385	8.55595203	0.95509926	27.5502227			23.9769184	27.1545639	7.66037023	2.04833012	16.32639389	0.17565713
1 h_2	82.80565467	22.4251086	26.5799984	19.6369346	3.7668592	13.6867071	6.274217869	10.27727373	70.7627802	60.9906	5.07559794	12.6157302	62.00194455	3.72578649
1 h_3	142.6080348	25.3253943	29.7782135	24.2909216	4.70256568	34.031474	6.11223995	9.805692686	67.8072899	53.4476391	7.35040833	3.66044644	31.43957265	0.37228066
1 h_4	7.096751736	1.05778624	1.27594187	0.99004823	0.73566035	1.01979001	1.066062106	1.501583292	1.94091311	2.22716493	0.88898944	0.43319368	9.355438751	0.23246101
1 h_5	2.884043763	0.6748289	0.79812431	0.55206083	0.21402619	0.43450215	1.101227464	1.169275362	0.9065547	0.98471558	0.39055053	0.19259829	3.531278416	0.15012796
1 h_6	7.720849048	1.00331368	1.09097806	0.81744562	0.40183979	1.0060165	1.166208172	1.604612507	2.1066592	2.57563644	0.87686022	0.48464084	8.644684156	0.22915123
2 h_1	102.2033027	28.1486347	22.9729096	18.5852905	3.03217716	58.130615			52.1751769	57.4233111	12.773316	5.01964414	48.39038899	0.62612042
2 h_2	124.886084	41.1670308	55.1611584	43.370623	10.8975833	16.9657537	6.128745864	12.95877916	118.513618	102.815492	7.92185073	21.4246429	95.1226066	8.19937328
2 h_3	153.9321692	31.0977781	38.0112758	31.365995	7.23832531	38.8943916	6.264234483	12.21646666	80.2271309	50.5219794	9.68986778	1.9468305	15.51322358	0.35619292
2 h_4	13.9997385	5.60991641	9.08138997	5.53927888	4.81913274	2.06120646	1.135434969	1.954174634	3.47331201	4.86207425	1.63634655	1.06167393	15.75363136	0.51792383
2 h_5	7.254819969	2.17171849	3.22779827	2.0279897	1.57762981	1.06704081	0.973862016	1.494723814	2.31110854	2.72889635	0.96305599	0.61045818	10.42797967	0.24668971
2 h_6	9.477525142	2.09557084	2.33879198	1.54447573	1.37193488	1.46665943	1.01576461	1.598096435	2.31241225	3.09682554	1.10356759	0.73609736	10.9699974	0.30811487
4 h_1														
4 h_2	131.7298346	39.0900594	50.0312918	40.3029718	8.96033444	22.9702324	5.783683795	12.3059182	115.160659	97.2898668	7.56226523	21.2978601	74.7906489	5.45760186
4 h_3	153.2482137	37.0627015	50.041558	40.4163342	10.4612243	43.0603352	5.792090758	10.76024473	99.2996114	77.300178	8.95060695	9.36787183	46.78468022	1.162878
4 h_4	12.75942363	1.85045297	2.48201522	1.94838652	1.04903041	1.80187248	1.080037526	1.742552087	4.09934522	4.1147104	1.21906357	0.72070176	11.58393529	0.32535383
4 h_5	21.50495671	4.00018709	5.07727575	3.76818554	2.79566041	3.01620203	1.173298665	1.932389193	6.40782343	6.89689705	1.82271958	1.36255168	22.01388731	0.52178493
4 h_6	7.036885051	1.43550477	1.89467571	1.43422329	0.79988668	1.17236718	1.143746075	1.595734522	2.0649623	2.69073133	0.79193632	0.5684671	8.736893836	0.47599104
6 h_1	169.1879083	61.4450126	63.5134412	41.8731061	5.71161463	90.6499145			110.080182	115.373835	18.679939	7.07959984	71.43039313	0.98867755
6 h_2	149.7540449	44.7087035	59.7099686	48.9924978	10.6929225	19.3325755	5.446566015	11.132173	142.425163	135.556929	5.91285607	27.120992	89.75918548	6.40006075
6 h_3	92.02859884	71.692002	102.431268	62.4498718	23.8088954	34.5865882	5.239460342	9.04634661	51.5981328	59.2866037	6.2627912	10.9140218	48.33881157	0.92399044
6 h_4	8.49977045	1.60893414	1.9874244	1.49158646	0.67217035	1.03507186	1.02953361	1.412792778	2.15357338	2.78417181	0.74158267	0.64195118	9.201124336	0.26582709
6 h_5	18.06728798	2.47029302	3.06757993	2.33127959	1.40976475	1.41330996	0.880683865	1.737606016	6.09251405	5.86106317	1.29432807	1.1799676	15.09710321	0.41040259
6 h_6	4.316561962	1.51850072	2.2902991	1.47315348	1.02296762	0.50289848	0.80638215	0.976933237	1.74284042	1.69300741	0.4477923	0.39015684	4.795330929	0.17194198
24 h_1	253.517088	67.6734107	59.1776964	42.2122979	9.78541543	108.036883			119.699841	119.096295	20.6551496	9.09871896	147.6237107	2.70102798
24 h_2	124.4779877	39.0383471	49.8823855	40.353536	10.6530934	14.9799416	5.278848913	10.05291188	79.9049342	73.7965243	6.34893999	14.3569567	78.83140209	4.9519445
24 h_3	159.4361512	38.7173541	50.3242129	41.6760546	12.5855306	39.5966279	5.400239681	10.04901805	81.5633705	71.784994	7.86274161	11.5604284	82.22867455	1.88752528
24 h_4	15.91113771	4.3195976	6.3952876	4.33043973	3.16641575	1.7830928	0.937180732	1.518184039	5.26077253	5.34696728	1.15329579	1.29478922	15.0271014	0.59987876
24 h_5	4.621677097	1.24173738	1.51234862	1.02950963	0.42144626	0.47583468	0.780863116	1.034423839	1.22999165	1.8337569	0.43247703	0.46791846	4.903690263	0.25183128
24 h_6	17.18433712	8.50049464	14.016842	8.66537193	7.71311624	1.97797473	0.855926546	1.893465165	7.72954628	7.59765382	1.38022006	1.63873292	19.51709194	0.69619847
48 h_1														
48 h_2	101.0071797	23.6327051	35.2801889	26.2910483	10.9974003	10.9527478	5.558258005	8.925370191	52.4273399	49.7053468	4.18738321	8.42361178	66.99879012	3.99384329
48 h_3	218.4093879	35.0827189	46.5392264	35.5086016	16.9252344	52.0132906	5.629741155	11.79554252	83.4430582	79.8229523	9.30836675	12.311498	147.7369045	3.14339783
48 h_4	26.94782892	6.83873545	8.5924152	5.97546283	4.13324006	1.91501336	1.068428558	1.903990296	6.84919294	8.45231575	1.18960164	1.32644831	15.74686619	0.52740933
48 h_5	31.57451241	5.23564606	5.68301334	4.43415754	2.06627976	1.27543034	1.062422061	2.158332641	8.62777144	10.2971416	1.61813274	1.62982755	20.62026943	0.852086
48 h_6	31.01147827	4.73202699	5.60936608	4.4597073	2.32410154	1.07449776	0.935918945	2.016896194	9.67334412	10.7273017	1.49407544	1.68500976	18.01380974	0.67970942

	Hydroxyglutarate	alpha-Ketoglutarate	Glutamic acid	Phenylalanine	Ornithine	(Iso)citric acid	Tyrosine	Glucose	Myoinositol	Tryptophan	Lysine
**control_1**		1.481532173	75.02991881	3.336466414		29.06574014	13.9541871	7.31380508	745.06328	1.56837445	
**control_2**	0.663273418	0.929837135	105.0907506	8.155869565	0.14363838	31.48887544	5.94612621	0.66362598	275.033326	2.43032729	
**control_3**	0.895203729	0.91331539	115.4567683	6.703557324	0.09815	28.45573696	9.68941622	0.63599908	479.69046	2.51937664	
**control_4**	0.08869127	0.087670324	10.53002174	0.654453846	0.0113115	2.574017853	0.97526103	0.0588021	21.5358258	0.31426779	0.04128664
**control_5**											
**control_6**											
**1 h_1**		0.874525322	1.713468427	0.692670637		12.93920064	4.91017922	1.54293086	240.09568	0.88693801	
**1 h_2**	0.939629981	1.205318004	118.5263226	14.26488793	0.21099612	38.58983418	11.770395	0.42831155	254.404863	5.06234702	
**1 h_3**	0.998370959	0.943037928	25.17690187	2.274230937	0.05588459	33.97066901	12.4273106	0.45180717	327.831604	1.56983371	
**1 h_4**	0.079400428	0.075940893	10.02511471	0.521945907	0.01526373	2.574956396	0.64005519	0.05401995	24.1073428	0.29202094	0.03630244
**1 h_5**	0.043231119	0.062665514	3.585106365	0.272550603	0.00797316	1.616924408	0.25387715	0.05021021	9.31510675	0.11903246	0.02464223
**1 h_6**	0.084232202	0.096578671	12.39669637	0.682853227	0.01352559	2.825480181	0.60787851	0.03900494	23.9975134	0.2047753	0.06627042
**2 h_1**		1.168924221	5.826970397	1.485865499		23.07839058	10.9959859	1.66195862	421.263776	1.36138324	
**2 h_2**	1.056040534	1.53052599	188.9792595	26.51270145	0.23169627	47.77805207	19.8770936	0.80984755	319.026459	7.16026815	
**2 h_3**	1.025365907	1.063196975	20.67417721	1.541182475	0.08043194	41.44618892	18.2038865	0.80583157	433.753378	3.04421442	
**2 h_4**	0.089414502	0.146739538	28.26238182	1.549688533	0.03195986	5.623302291	1.23421279	0.10065053	48.2670426	0.57422444	0.21660018
**2 h_5**	0.078434624	0.076010474	16.43421435	0.942469037	0.0152514	2.825529937	0.64114216	0.03888084	27.6095091	0.31388303	0.13572061
**2 h_6**	0.086163843	0.135177964	21.58536851	1.204979417	0.02632103	5.318585322	0.74442375	0.05243417	34.2503165	0.38984257	0.18491371
**4 h_1**											
**4 h_2**	0.952244772	1.286994219	153.7329096	24.83423911	0.27367595	47.67942111	25.6093586	0.83326314	344.078166	7.37063509	
**4 h_3**	0.860667505	1.021593341	56.86769599	5.429883728	0.15236617	34.6274072	25.9385764	0.837183	363.185891	4.2553842	
**4 h_4**	0.077760514	0.094733684	16.80646924	0.985730061	0.021761	3.554062913	1.22538145	0.05308938	25.8253358	0.38231228	0.1003137
**4 h_5**	0.106403476	0.142524551	36.45205645	1.988305175	0.06415652	4.657450013	2.24954817	0.08589315	48.541091	0.78945153	0.23856043
**4 h_6**	0.067299839	0.093956226	14.38904379	0.962417095	0.03062466	3.42710558	1.07437076	0.07011953	20.0651033	0.45309675	0.10903428
**6 h_1**		1.337447092	11.99688107	1.74708967		34.18664293	20.2782417	2.34073117	605.219785	2.06071084	
**6 h_2**	0.743094315	1.022562573	158.121151	31.64373514	0.26729271	38.18131164	24.7673972	0.97436702	252.191775	8.47610658	
**6 h_3**	0.741888256	0.760992577	59.65754799	7.69568586	0.15249779	23.39441147	17.7516597	0.75121892	243.49534	3.45541105	
**6 h_4**	0.049809581	0.091425684	15.04084536	1.006521907	0.01934744	3.010610437	0.78702291	0.04269812	18.2384419	0.36924018	0.12707087
**6 h_5**	0.070987981	0.132177945	28.81395561	1.725399898	0.03352028	3.397813393	1.33335987	0.06190763	30.5926377	0.61501402	0.20085269
**6 h_6**	0.046289866	0.067571916	8.926150131	0.569338667	0.01749262	1.82289292	0.34722313	0.04185043	9.62185441	0.20053034	0.09533555
**24 h_1**		1.220537966	33.29620818	3.962849098		33.99229108	30.9476146	3.83787356	657.777795	4.41659678	
**24 h_2**	0.629160814	0.738002039	112.4907222	21.92142108	0.30590268	35.99950612	17.691173	0.91748175	204.524558	6.34833252	
**24 h_3**	0.687773046	0.805972575	93.50726422	13.58971417	0.2458873	24.0072565	24.968994	1.21352598	291.428653	5.60460802	
**24 h_4**	0.06120579	0.112989548	25.08262848	1.988193905	0.05630189	4.492998981	1.48735476	0.08864149	30.6986393	0.78581047	0.36367082
**24 h_5**	0.032534334	0.071296128	8.177845535	0.740018001	0.01660452	1.812999241	0.39072898	0.0878533	9.93410607	0.30474998	0.12564267
**24 h_6**	0.061380461	0.127959773	30.47446518	2.562543528	0.06568598	4.440443488	1.01809013	0.10508835	35.7251538	1.0201991	0.5985944
**48 h_1**											
**48 h_2**	0.681761424	0.603237206	75.64526093	13.98787112	0.28194681	29.39692984	9.09920945	1.39837692	170.349296	4.35403274	
**48 h_3**	0.749518708	0.875127895	165.0883635	18.43543643	0.53567632	32.02165149	29.7498713	1.7029747	560.322616	7.79296106	
**48 h_4**	0.061536773	0.108048874	23.26122421	1.995036867	0.02962256	5.624638296	3.21948992	0.09630936	39.4916001	0.70909885	0.10929763
**48 h_5**	0.078036846	0.113790855	32.32729576	2.601277782	0.02336041	8.680408444	4.32736251	0.13769877	46.2679174	1.06743222	0.11574465
**48 h_6**	0.080761793	0.095769324	29.89527923	2.672736937	0.02152871	6.521220453	4.50410418	0.11461068	40.1320361	1.20807787	0.07705603

Replicates 1 to 3 and 4 to 6 were measured in the same experiment, respectively. Data was normalized to control (100 %) for comparison. 1 to 3 was normalized to respective control, 4 to 6 were normalized to control_4. Not every metabolite was detected in every replicate and respective values were left blank. For replicate 4 to 6, 2 technical replicates were performed, here the mean value is depicted.

Table S2. Raw data for targeted metabolite abundances measured via GC-MS from human astrocytoma cells treated with 5 mM NH_4Cl and harvested immediately afterwards (0 h).

	Glutamic acid
**control_1**	0.1904
**control_2**	0.2758
**control_3**	0.4481
**0 h_1**	3.0199
**0 h_2**	3.0735
**0 h_3**	2.6139

Table S3. Summary of quantification method for ^{15}N-labeled amino acids measured via LC-QTOF. Shown are all theoretically possible isotopomers after ^{15}N-labeling. Isotopomer masses colored in red could not be determined in any sample due to sensitivity or absence in the sample. Natural abundances were retrieved from MassHunter Isotope Distribution Calculator version B7024.29.

			Mass of isotopomer (m/z)					Natural abundance of isotopomer (%)				
**Compound**	**Formula**	**Retention time (min)**	**m0**	**m1**	**m2**	**m3**	**m4**	**m0**	**m1**	**m2**	**m3**	**m4**
alpha-Alanine	C3H7NO2	2.92	90.05495	91.05779				95.91521	3.62391			
Arginine	C6H14N4O2	20	175.11895	176.12125	177.12328	178.12547	179.12755	91.7848	7.52586	0.65109	0.03661	0.00158
Asparagine	C4H8N2O3	3.39	133.06077	134.06332	135.06512			94.30294	4.97424	0.68922		
Aspartate	C4H7NO4	2.2	134.04478	135.04775				94.42833	4.66098			
Cysteine	C3H7NO2S	2.58	122.02703	123.02926				91.10993	4.16172			
Glutamate	C5H9NO4	2.66	148.06043	149.06348				93.39646	5.64169			
Glutamine	C5H10N2O3	3.63	147.07642	148.07912	149.08089			93.27244	5.95014	0.73626		
Glycine	C2H5NO2	2.5	76.0393	77.0419				96.97491	2.59279			
Histidine	C6H9N3O2	14.36	156.07675	157.07927	158.08125	159.08356		92.1731	7.16796	0.62333	0.03412	
Isoleucine	C6H13NO2	6.2	132.10191	133.10501				92.8051	6.58171			
Leucine	C6H13NO2	5.72	132.10191	133.10501				92.8051	6.58171			
Lysine	C6H14N2O2	11.37	147.1128	148.11562	149.11753			92.45666	6.9054	0.60417		
Methionine	C5H11NO2S	4.2	150.05833	151.06095				89.12962	6.04027			
Phenylalanine	C9H11NO2	6.56	166.08626	167.08943				89.87849	9.26979			
Proline	C5H9NO2	2.88	116.0706	117.07364				93.85202	5.5977			
Serine	C3H7NO3	2.32	106.04987	107.05272				95.68214	3.65155			
Threonine	C4H9NO3	2.6	120.06552	121.06849				94.63657	4.65698			
Tryptophan	C11H12N2O2	11.13	205.09715	206.10019	207.10256			87.63511	11.26432	1.02654		
Tyrosine	C9H11NO3	4.2	182.08117	183.08435				89.66009	9.28141			
Valine	C5H11NO2	4	118.08626	119.0893				93.83044	5.618			

Disease Models & Mechanisms • Supplementary information

Table S4. Raw data from quantification method for ^{15}N-labeled amino acids measured via LC-QTOF.

rel. enrichment m+1	alpha-Alanine	Aspartate	Glutamate	Histidine	Isoleucine	Leucine	Proline	Valine
**control_1**	0	0.40090837	0.47125559	0.91527038	0	0.05577544	0	0.20031903
**control_2**	0	0.48510137	1.19260657	0.37448333	0	0.80310617	0.34308631	0
**control_3**			1.29267167	0	0	0	0	0
**1 h_1**	1.04923406	7.42071568	7.30439269	2.4884458	1.10860703	1.10742484	3.82161783	0
**1 h_2**	1.1342786	5.57061267	8.08730538	1.33380341	0.5122767	0.16596954	4.38318515	0.54090537
**1 h_3**	1.4235847	11.7719037	7.05797965	2.94279624	1.56580823	1.87013479	3.25527612	0
**2 h_1**	1.02353373	4.94454043	10.6995843	0	1.6910971	2.21479582	7.38352294	0.94699479
**2 h_2**	1.50847976	11.6258644	9.14597736	0.15150108	2.01387955	4.44076122	6.13227587	0.77000518
**2 h_3**	1.63380619	7.96397967	10.0765488	3.40861241	1.21635719	1.44060611	6.73474321	0.31476989
**4 h_1**	2.77573225	17.8409345	22.2289823	0.73633046	2.64423697	4.70825532	15.2264821	1.21290403
**4 h_2**	1.58387165	15.1567539	19.8113117	0.27736634	2.20542332	4.1037447	13.5017598	1.15444201
**4 h_3**	3.25053851	17.4705266	22.8746761	0	3.05079323	5.74084546	15.1134947	1.43422227
**6 h_1**	1.48273715	11.1146972	15.724708	0.07174415	2.13343836	3.26234773	12.6294388	1.31192073
**6 h_2**	1.04540805	10.6964563	14.9846617	0.40007852	2.37162829	4.13902977	11.8173998	1.51042917
**6 h_3**	1.81965465	15.2847053	18.0640338	0	3.12946529	4.56325965	14.8371646	1.8754336
**24 h_1**	1.91397889	15.4363329	20.3238226	0.52366814	2.48802449	3.60320655	18.6531695	1.21526608
**24 h_2**	2.22740472	16.250518	20.3090225	0	2.99205761	5.24440308	18.3253367	1.25429893
**24 h_3**	2.47775372	16.5117326	18.2038927	1.00092673	3.74397819	5.57323623	17.2402266	2.17686156
**48 h_1**	1.50324848	8.96337872	12.0635386	0.29616694	2.46188169	3.6951194	11.187649	0.76294907
**48 h_2**	1.31638932	9.8710555	13.2015612	0	2.05882482	3.43128094	12.2095382	0.93567938
**48 h_3**	1.2572175	10.4702217	14.2606943	0.48794577	2.39205954	3.96856915	11.7648981	3.40078421

Shown are only metabolites with a relative enrichment of ^{15}N of at least 1 % at any time point.

Data analysis for ^{15}N-labeled amino acids. Amino acid peaks in the samples were identified at mass-to-charge (m/z) ratios and retention times (RT) listed above and with external amino acid standards measured in parallel. Peaks were integrated via Agilent Mass Hunter Workstation B07 (Agilent Technologies, Santa Clara CA, USA). Peak intensities of m1 (i.e. incorporation of one ^{15}N-label) were corrected for their natural abundance via the following calculation.

$m0_{int}$, peak intensity of non-labelled amino acid; $m1_{int}$, peak intensity of amino acid with one ^{15}N-label (m+1); $m1_{nat}$, natural abundance of isotopomer (see above);

$m1_{background} = m0_{int} \bullet m1_{nat} : 100$; $m1_{enrichment} = m1_{int} - m1_{background}$; $m1_{relative.enrichment} = m1_{enrichment} : m0_{int} \bullet 100$

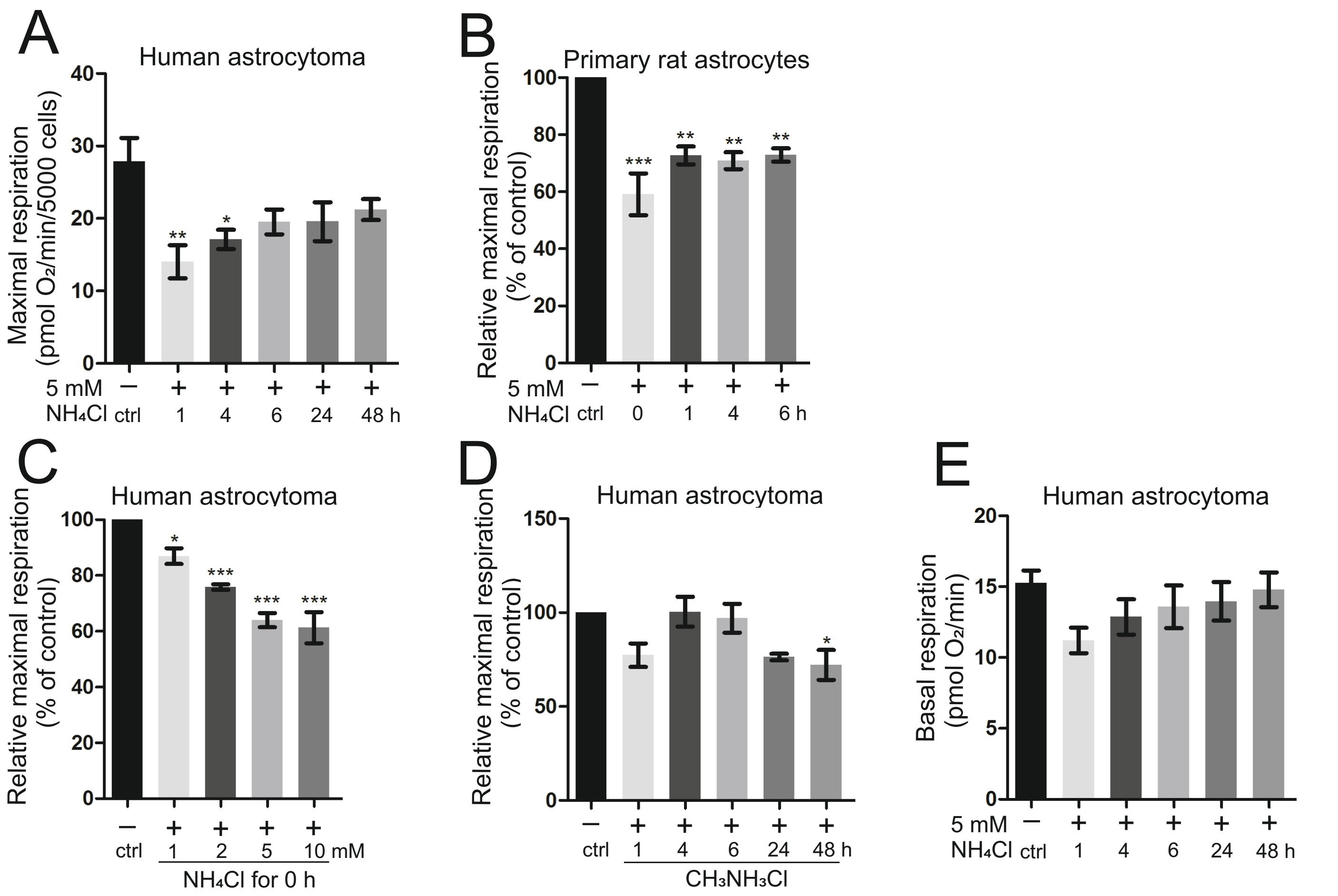

Figure S1: Mitochondrial respiration is immediately impaired by ammonia in a pH-independent manner. Oxygen consumption rate (OCR) of human astrocytoma cells and primary rat astrocytes was analyzed in Seahorse XFe96 Extracellular Flux Analyzer with the Mito Stress Test Kit after treatment with ammonia at indicated molarities or durations. (A) Maximal respiration of human astrocytoma cells after treatment with 5 mM NH_4Cl for 1-48 h (n=5-7). (B) Relative maximal respiration of primary rat astrocytes after treatment with 5 mM NH_4Cl for 1-6 h and directly after treatment (0 h) (n=3-4). (C) Relative maximal respiration of human astrocytoma cells and directly after treatment (0 h) with 1, 2, 5, or 10 mM NH_4Cl (n=3). (D) Relative maximal respiration of human astrocytoma cells was determined after treatment with 5 mM CH_3NH_3Cl (pH-mimetic) for 1-48 h (n=3). (E) Basal respiration of human astrocytoma cells treated with 5 mM NH4Cl for 1-48 h (n=8). (B), (C), (D) Individual biological replicates normalized to control (100%) are depicted. Data presented as mean ± SEM. Statistics: One-way ANOVA with Dunnett's post test (all treatments vs. control). $^*P < 0.05$, $^{**}P < 0.01$, $^{***}P < 0.001$.

Disease Models & Mechanisms • Supplementary information

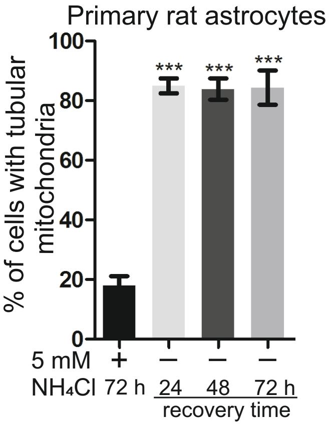

Figure S2: Ammonia-induced mitochondrial fragmentation is reversible. Primary rat astrocytes transfected with pEGFP-Mito. Baseline quantification was performed after 72 h treatment with 5 mM NH_4Cl. Other time points represent recovery time periods after removal of ammonia. Characterization of mitochondria with respect to fragmented versus tubular morphology. Data represented mean ± SD (n=3). One-way ANOVA with Dunnett's post test (all treatments vs. control). ***P < 0.001.

Disease Models & Mechanisms • Supplementary information

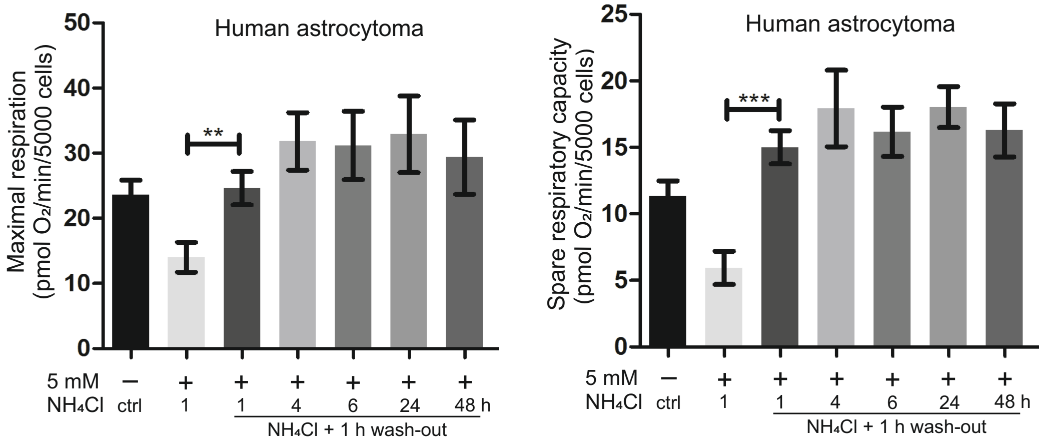

Figure S3: Ammonia-induced decrease in oxygen consumption rate is rapidly reversible. Human astrocytoma cells were analyzed using the Mito Stress Test Kit on Seahorse XFe96 Extracellular Flux Analyzer. Oxygen consumption rate (OCR) of maximum respiration (left) and spare respiratory capacity (right) were determined after 1 h wash-out of ammonia treated with 5 mM NH_4Cl for 1-48 h and compared to 1 h treatment 5 mM NH_4Cl. Data represented as mean ± SEM (n=3). Student's t-test, one-tailed, unpaired. ** $P < 0.01$, *** $P < 0.001$.

Disease Models & Mechanisms • Supplementary information

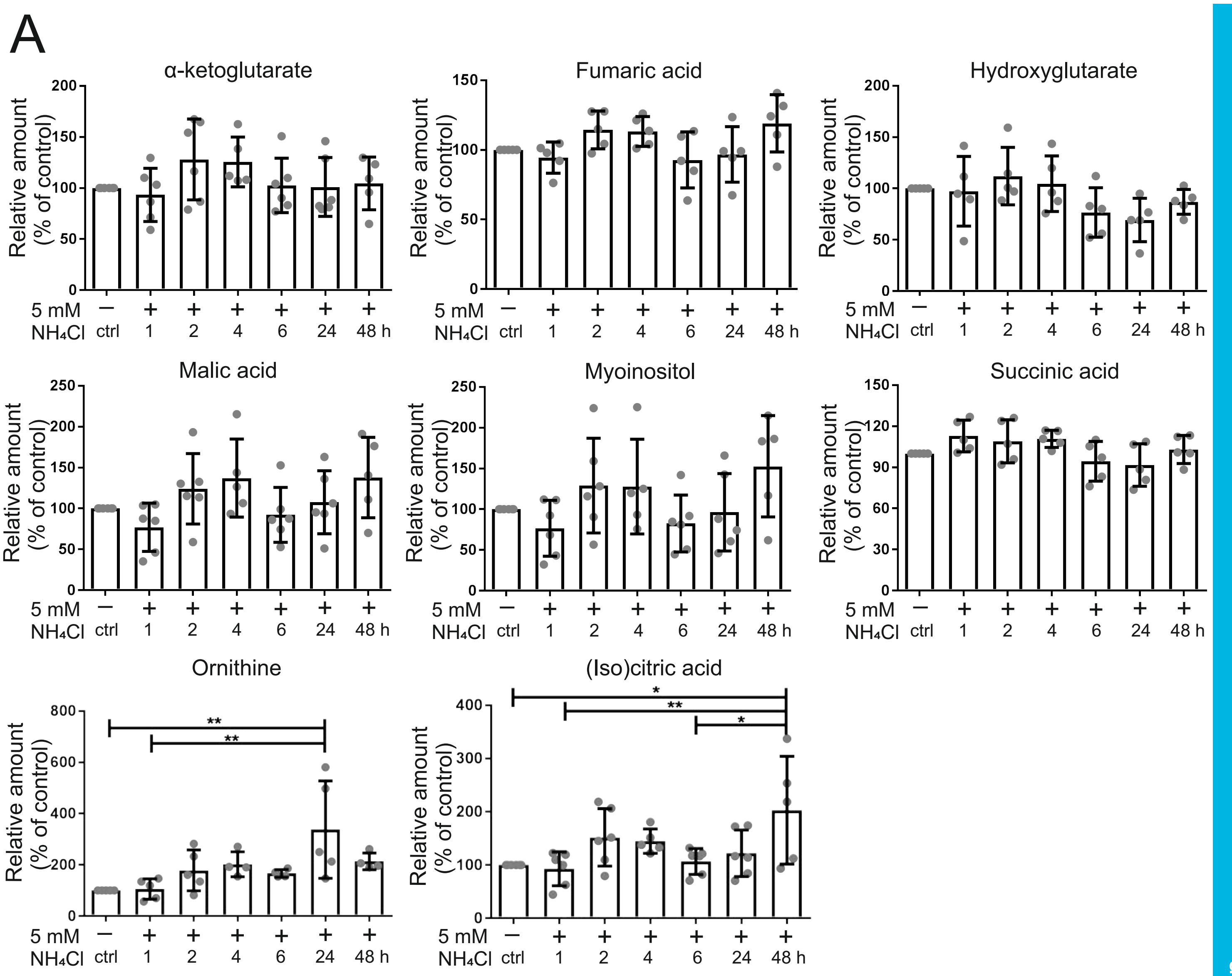

Disease Models & Mechanisms • Supplementary information

Figure S4A

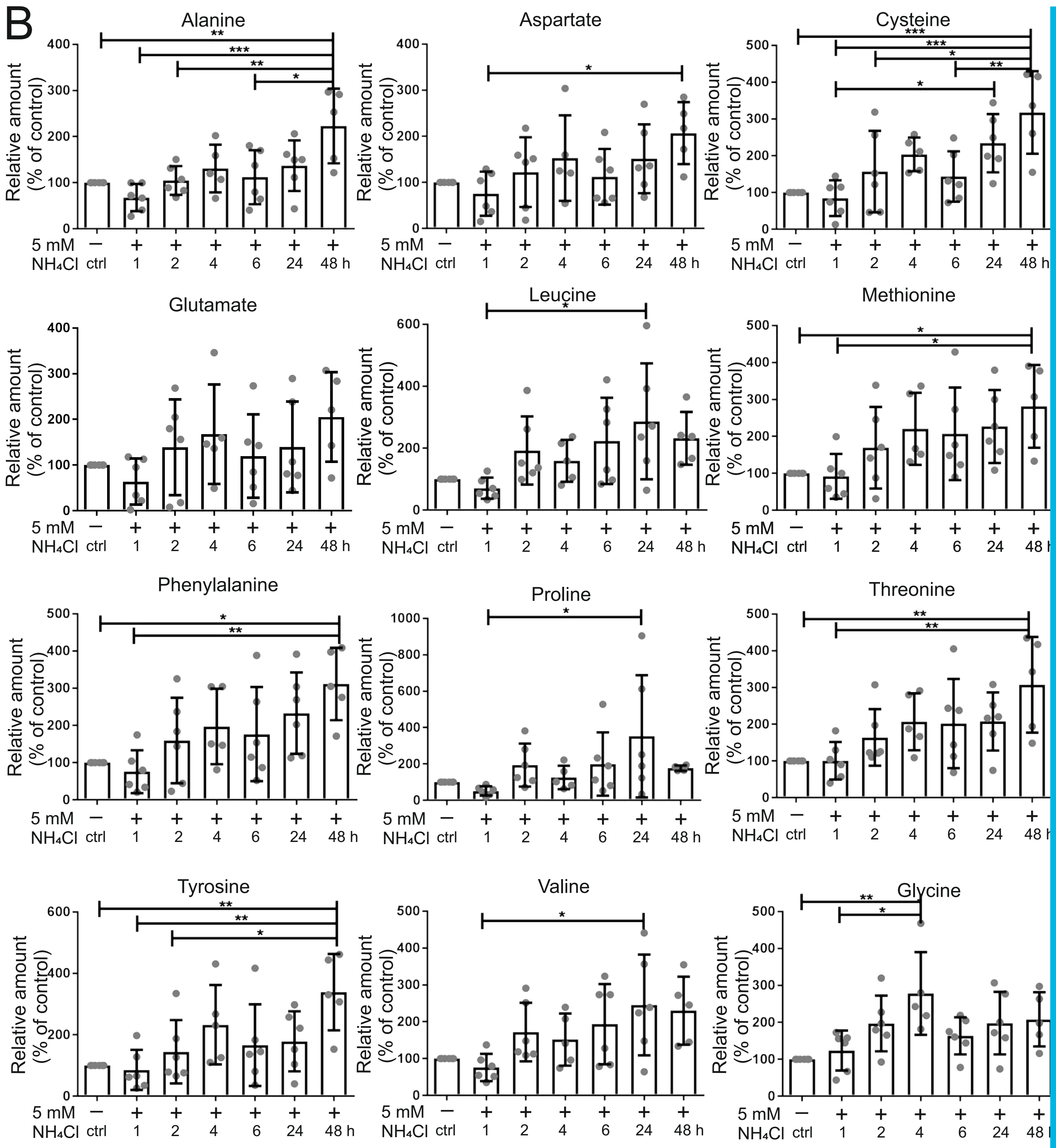

Figure S4: Extended data from steady-state metabolomics. Mass Spectrometry for steady-state metabolites was done in human astrocytoma cells on GC-QTOF. Treatment with 5 mM NH_4Cl for 1-48 h. Relative abundance of respective metabolites compared to controls (100 %) over time. (A) Additional non-amino-acid metabolites and details not shown in the heat map of Fig. 4A or Fig. 4B. (B) Additional details for detected amino acids shown in the heat map of Fig. 4A or Fig. 4B. Data represent mean ± SD (n=4-6). Statistics: One-way ANOVA with Tukey's post test (all samples vs. all samples). *P < 0.05, **P < 0.01, ***P < 0.001.

Figure S4B

Disease Models & Mechanisms • Supplementary information

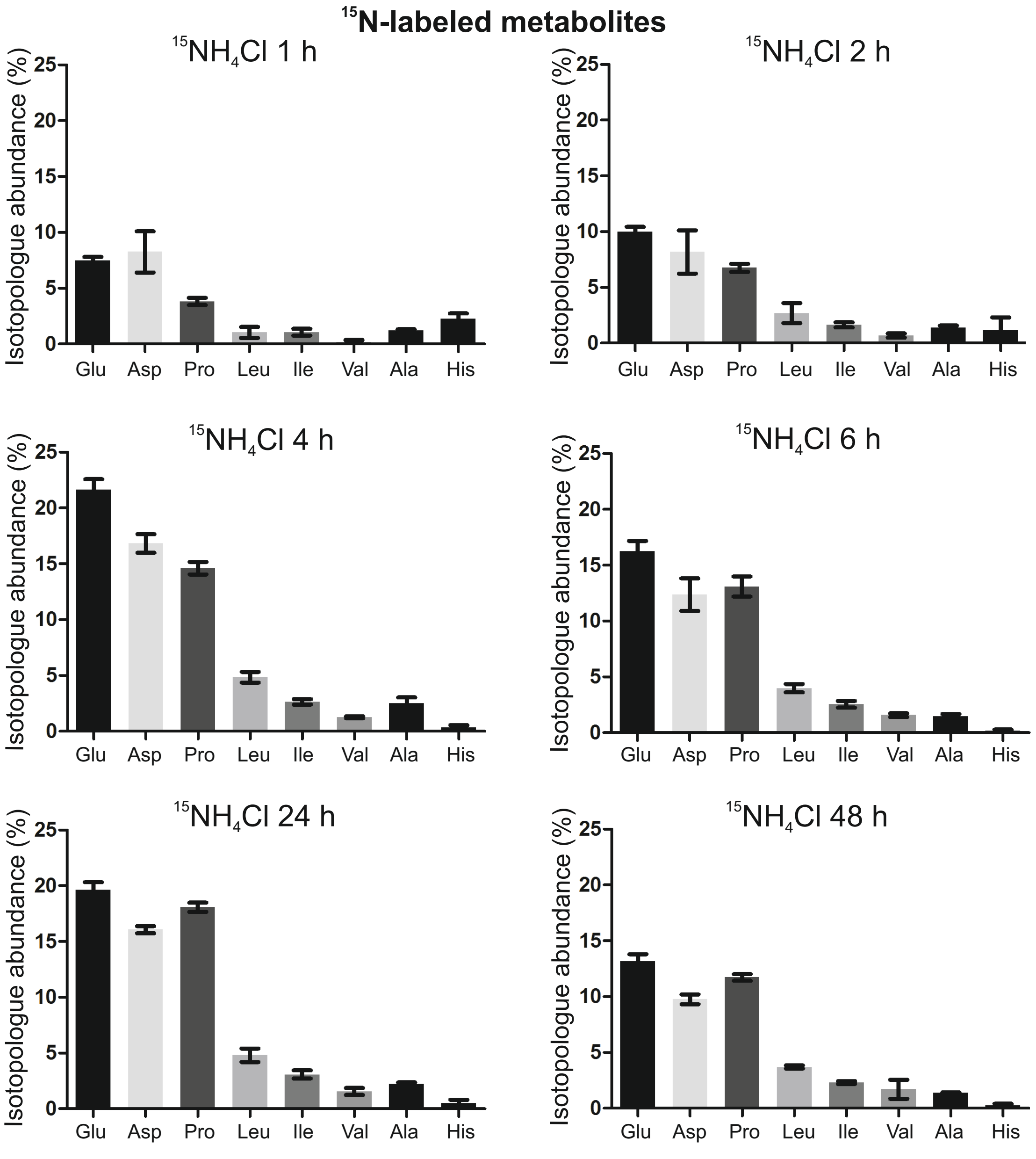

Figure S5: ^{15}N-isotopologue abundance of labeled amino acids over time. Mass Spectrometry for ammonia flux was done in human astrocytoma cells on LC-QTOF. Cells were treated with $^{15}NH_4Cl$ for 1-48 h and isotopologue abundance of ^{15}N in Glu, Asp, Pro, Leu, Ile, Val, Ala and His was determined over time. Data represent mean ± SD (n=3).

Disease Models & Mechanisms • Supplementary information

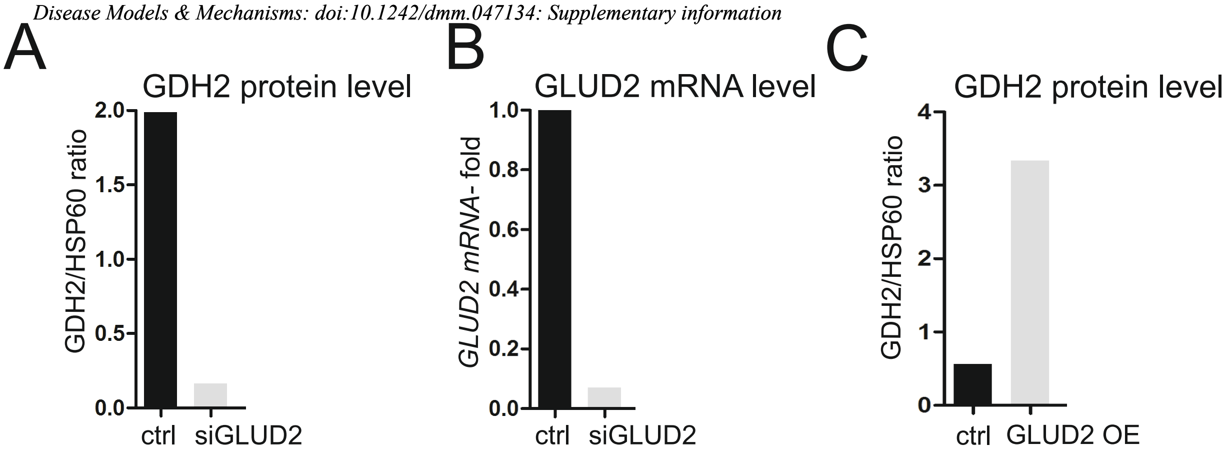

Figure S6: Protein and/or mRNA level of glutamate dehydrogenase after *GLUD2* knock-down and overexpression. *GLUD2* knock-down validation in human astrocytoma cells. (A) Densitometry of protein levels determined by Western blot analysis (Fig. 6A) using HSP60 as loading control. Ratios of GDH2 to HSP60 levels are shown in knock-down *vs*. control cells. (B) mRNA level was determined by qPCR using HPRT1 as housekeeping gene. *GLUD2* expression levels are depicted in knock-down cells as compared to control and normalized to 1. (C) Densitometry of protein levels determined by Western blot analysis (Fig. 6A) to validate overexpression (OE) of *GLUD2* normalized to loading control HSP60. Representative experiments are shown.

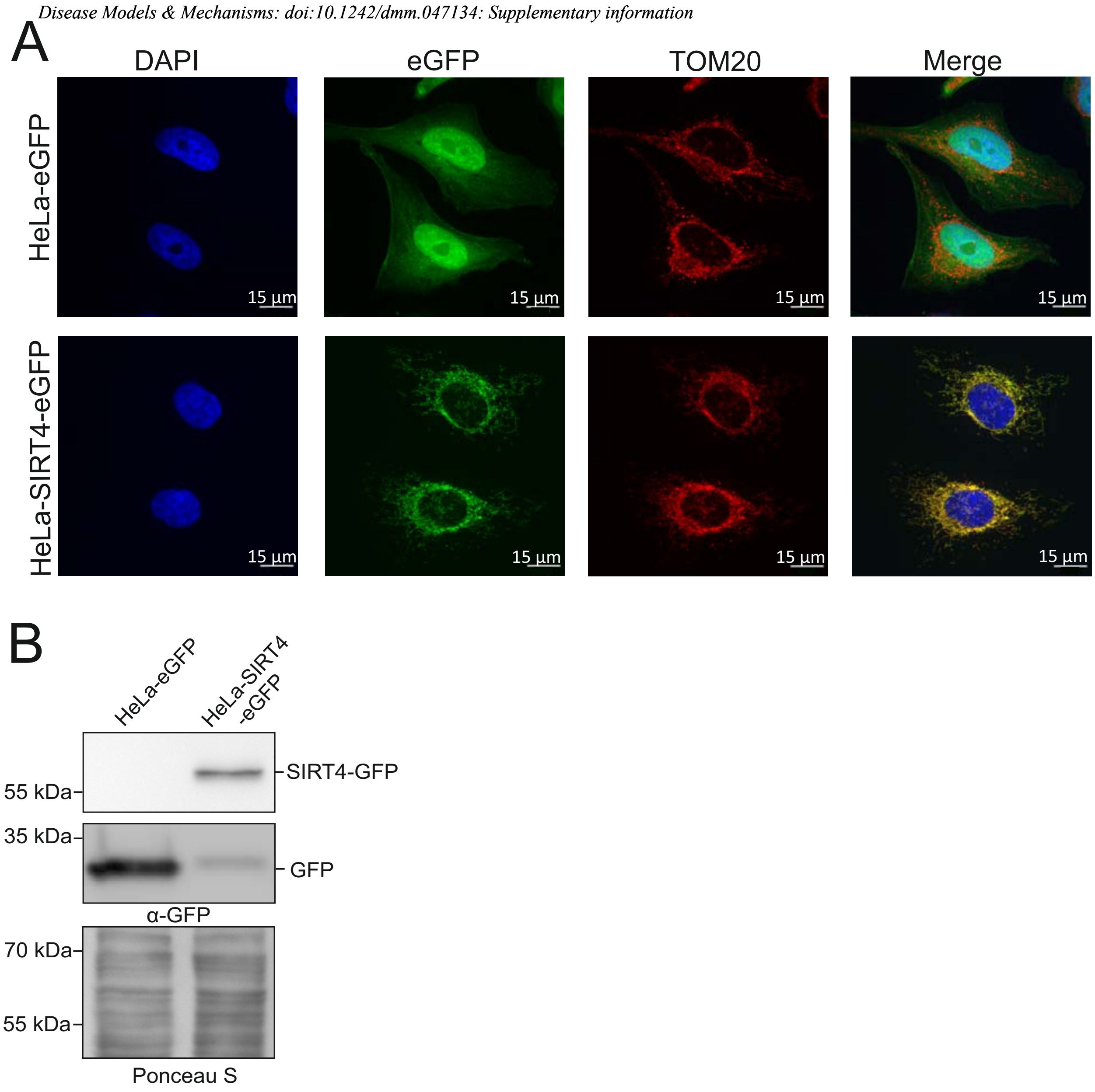

Figure S7: Expression of SIRT4 and localization to mitochondria. (A) Representative images showing the localization of SIRT4-GFP to mitochondria. Immunostaining against TOM20 (mitochondria) and DAPI (nucleus) staining in HeLa-eGFP and HeLa-SIRT4-eGFP cells. (B) Western blot showing the overexpression of SIRT4-eGFP construct in HeLa-eGFP vs. HeLa-SIRT4-eGFP cells. Ponceau S staining to control for loading.

Disease Models & Mechanisms • Supplementary information

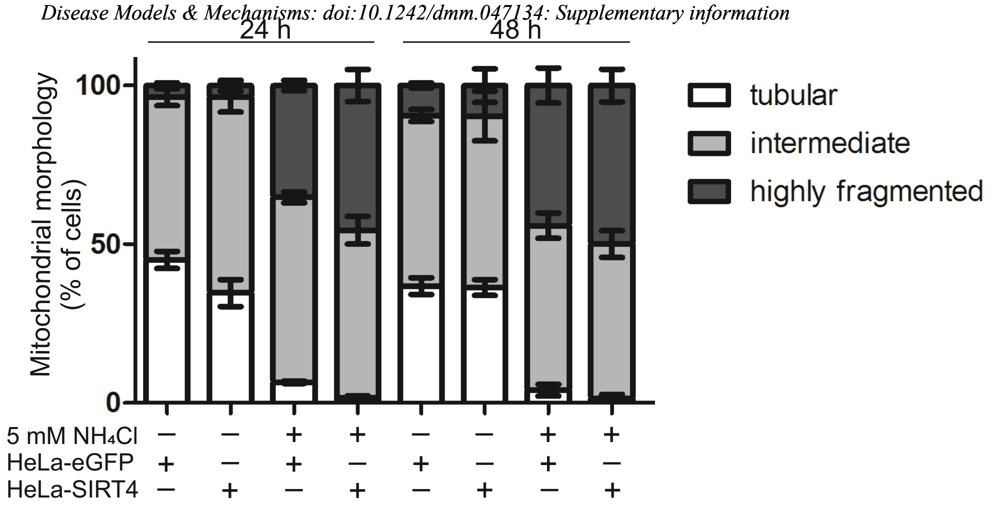

Figure S8: SIRT4 overexpression does not restore ammonia-induced changes in mitochondrial morphology. Mitochondria in HeLa-eGFP and HeLa-SIRT4-eGFP cells were visualized by immunostaining against TOM20. Cells were treated with 5 mM NH_4Cl for respective duration 24 or 48 h. At least 20 pictures were taken per sample showing approximately 10-15 cells each. Mitochondria were categorized to tubular, intermediate and fragmented morphological phenotype and morphological changes are depicted over time. Percentage of cells with respective phenotype are shown. Data presented as mean ± SEM (n=3).

Disease Models & Mechanisms • Supplementary information

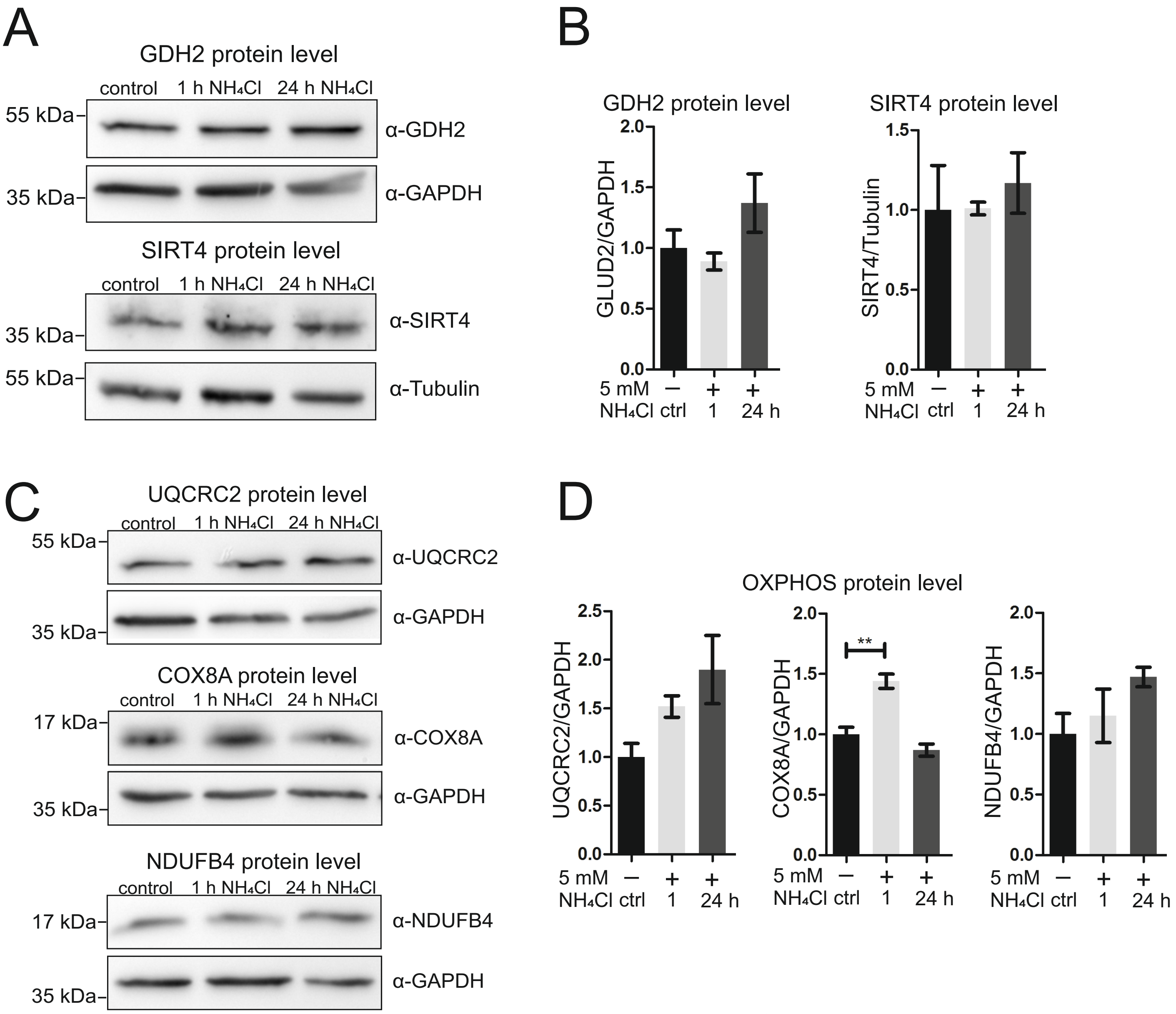

Figure S9: Changes in protein levels of GDH2, SIRT4 and OXPHOS enzymes. (A) Representative Western blots of GDH2 and SIRT4 protein levels after treatment with 5 mM NH_4Cl for 1 or 24 h compared to control. GAPDH and Tubulin are used as a loading control, respectively. (B) Densitometry of Western blots shown in A corrected to respective loading control and normalized to control. (C) Representative Western blots showing protein levels of UQCRC2 (complex III), COX8A (complex IV) and NDUFB4 (complex I) after treatment with 5 mM NH_4Cl for 1 or 24 h compared to control. GAPDH is used as a loading control. (D) Densitometry of Western blots shown in C corrected to GAPDH and normalized to control. Data presented as mean ± SEM (n=3). Statistics: One-way ANOVA with Dunnett's post test (all treatments vs. control). **$P < 0.01$.

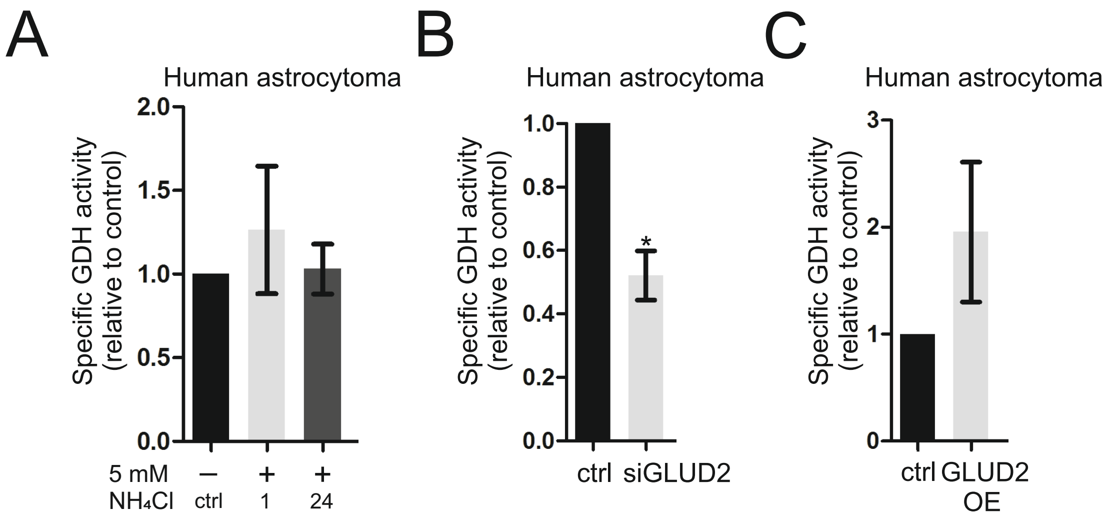

Figure S10: Ammonia treatment alone does not grossly affect specific GDH activity. (A) Human astrocytoma cells were treated with 5 mM NH_4Cl for 1 or 24 h and GDH activity was measured in 1 x 10^6 cells. (B) *GLUD2* knock-down was done in human astrocytoma cells using a GDH-targeting siRNA for 48 h. Knockdown is validated by reduction in GDH activity. (C) GDH2 was overexpressed in human astrocytoma cells for 48 h. Overexpression of GDH2 is corroborated by increased GDH activity. GDH activity was determined by a commercial assay detecting NADH production. Values were individually normalized to control and normalized to total protein content using a Bradford assay. Data is presented as mean ± SEM (n=3). Statistics: One-sample t-test. * $P < 0.05$.

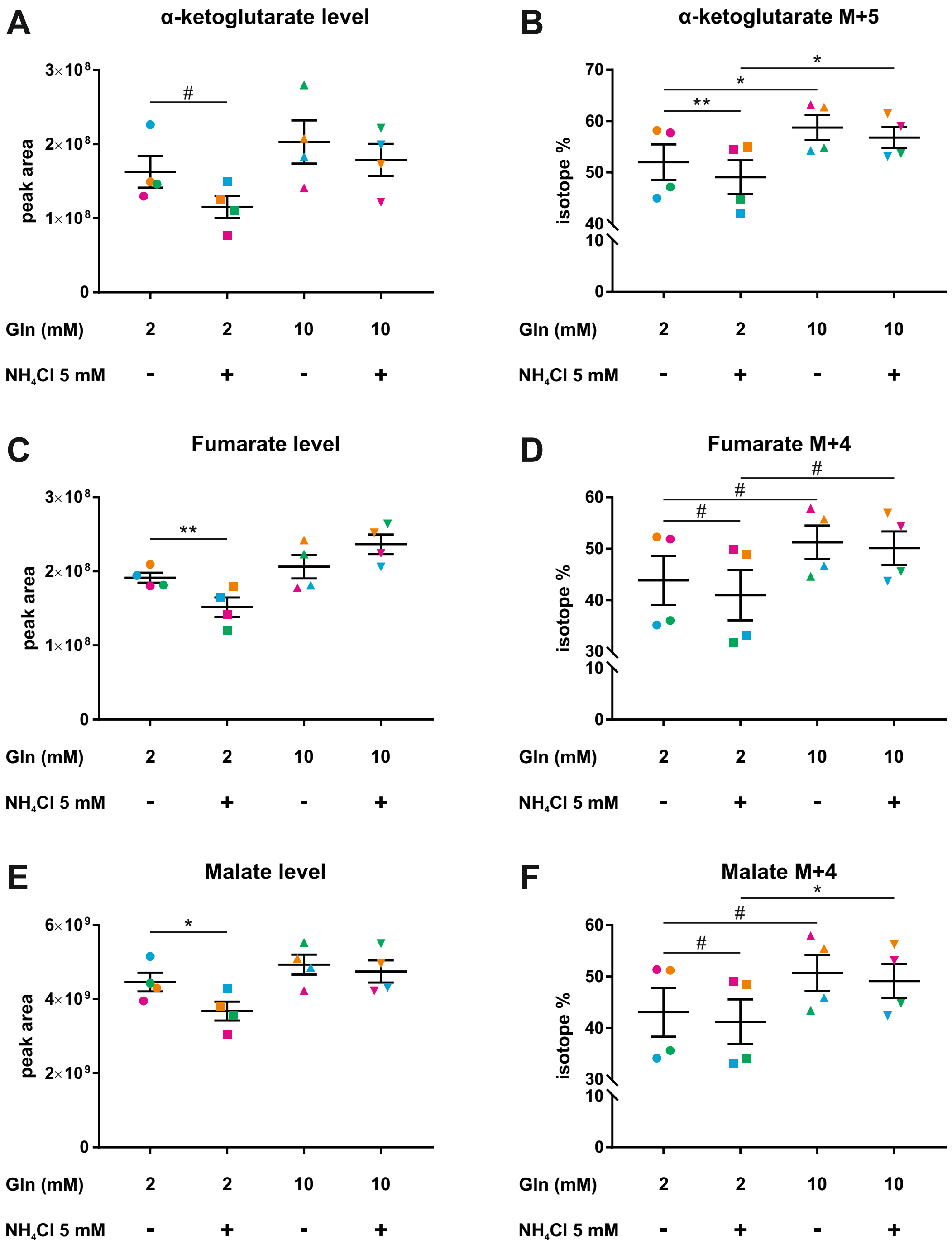

Figure S11. Ammonia reduces the influx of carbon into the TCA-cycle from isotope labeled glutamine. MOG-G-CCM cells were grown to confluency and were exposed to fresh growth medium containing 2 or 10 mM ^{13}C-labeled glutamine and 0 or 5 mM NH_4Cl for 40 minutes before cell harvest. The levels of aKG, fumarate and malate as well as their isotope distributions were analyzed via IC-MS in 4 independent experiments. Results are shown as individual experiments in different colors and mean ±SEM. Statistical analysis was conducted with paired one-way ANOVA with Geisser-Greenhouse correction and Sidak's multiple comparison post-hoc test comparing 2 mM Gln vs. 2 mM Gln + NH_4Cl, 2 mM Gln vs. 10 mM Gln, 2 mM Gln + NH_4Cl vs. 10 mM Gln + NH_4Cl, and 10 mM Gln vs. 10 mM Gln + NH_4Cl. Only significant comparisons are indicated (# P<0.1, * P<0.05, ** P<0.01). At 10 mM Gln addition of ammonia shows no significant alterations of TCA-cycle intermediates.

Figure S11

Disease Models & Mechanisms • Supplementary information
